# Verrucosamide, a Cytotoxic 1,4-Thiazepane-Containing Thiodepsipeptide from a Marine-Derived Actinomycete

**DOI:** 10.3390/md18110549

**Published:** 2020-11-05

**Authors:** Vimal Nair, Min Cheol Kim, James A. Golen, Arnold L. Rheingold, Gabriel A. Castro, Paul R. Jensen, William Fenical

**Affiliations:** 1 Center for Marine Biotechnology and Biomedicine, Scripps Institution of Oceanography, University of California, San Diego, CA 92093-0204, USA; vimal.nair16@gmail.com (V.N.); mck008@ucsd.edu (M.C.K.); g8castro@ucsd.edu (G.A.C.); pjensen@ucsd.edu (P.R.J.); 2 Department of Chemistry and Biochemistry, University of California, San Diego, CA 92093, USA; jgolen@umassd.edu (J.A.G.); arheingold@ucsd.edu (A.L.R.); 3 Skaggs School of Pharmacy and Pharmaceutical Science, University of California, San Diego, CA 92093, USA; 4 Moores Comprehensive Cancer Center, University of California, San Diego, CA 92093, USA

**Keywords:** marine actinomycetes, cytotoxic thiodepsipeptides, 1,4-thiazepane

## Abstract

A new cytotoxic thiodepsipeptide, verrucosamide (**1**), was isolated along with the known, related cyclic peptide thiocoraline, from the extract of a marine-derived actinomycete, a *Verrucosispora* sp., our strain CNX-026. The new peptide, which is composed of two rare seven-membered 1,4-thiazepane rings, was elucidated by a combination of spectral methods and the absolute configuration was determined by a single X-ray diffraction study. Verrucosamide (**1**) showed moderate cytotoxicity and selectivity in the NCI 60 cell line bioassay. The most susceptible cell lines were MDA-MB-468 breast carcinoma with an LD_50_ of 1.26 µM, and COLO 205 colon adenocarcinoma with an LD_50_ of 1.4 µM. Also isolated along with verrucosamide were three small 3-hydroxy(alkoxy)-quinaldic acid derivatives that appear to be products of the same biosynthetic pathway.

## 1. Introduction

Marine-derived actinomycete bacteria have been investigated for novel molecules over the past three decades and have been recognized as a significant source for drug leads reported to possess a wide scope of activities [1,2,3,4,5]. The new molecule described here, verrucosamide (**1**), is closely related to several previously reported metabolites, the classic example is BE-22179 (**2**), a thiodepsipeptide reported in 1994 from the soil-derived actinomycete *Streptomyces* A22179 isolated in Japan [6]. Subsequent to this discovery, the related metabolite thiocoraline (**3**), produced by the marine-derived actinomycete *Micromonospora* sp. was discovered [7,8] and more recently, a series of thiocoraline analogs was reported from several strains of the marine-derived bacterium *Verrucosispora* sp. [9]. These examples are characterized by the presence of a 3-hydroxyquinaldic acid chromophore coupled with a thiodepsipeptide core. More distantly related are the thiodepsipeptides triostin A and echinomycin containing quinoxaline (1,4-diazanaphthalene) chromophores [10,11]. These two agents have been shown to unwind negatively supercoiled double-stranded DNA and to bind to DNA by bisintercalation [12,13]. The bioactivity of these two agents, along with the interesting activity of thiocoraline, created considerable interest in this class of cyclic thiodepsipeptides.

**Figure 1:**
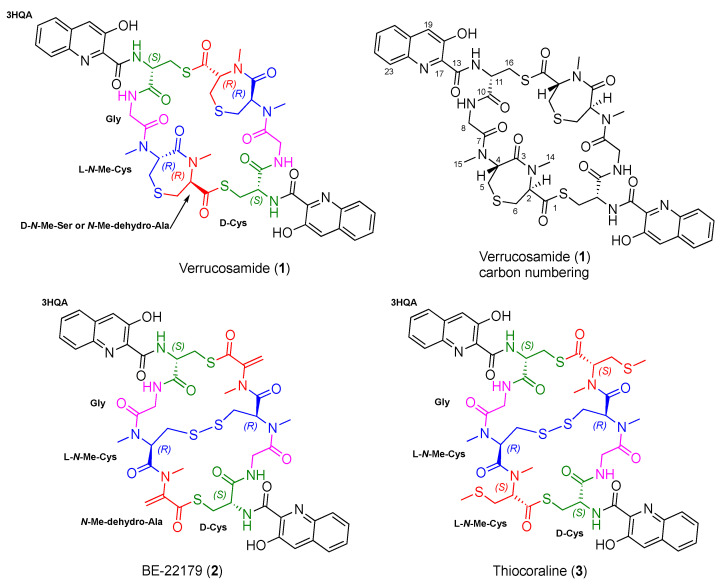
Chemical structure of verrucosamide (**1**) and the related thiodepsipeptides BE-22179 (**2**) and thiocoraline (**3**).

**Figure 2:**
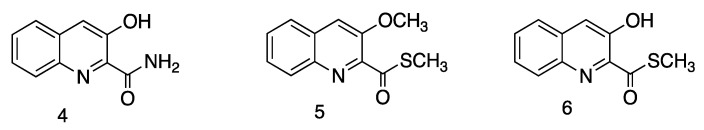
Small 3-hydroxy(alkoxy)-quinaldic acid derivatives isolated from the culture of *Verrucosispora* sp., strain CNX-026.

In a continuing effort to identify marine bacterial agents with potential in the treatment of cancer [14,15,16], extracts from a cultured *Verrucosispora* sp. (our strain CNX-026) were screened against the HCT-116 human colon carcinoma cell line. The crude extract showed significant HCT-116 activity which suggested a thorough study. After purification and compound isolation, testing showed that the cytotoxicity activity could be assigned to thiocoraline (**3**) and a new thiodepsipeptide verrucosamide (**1**). The strain also produced three other lower molecular weight molecules **4**–**6** having a similar 3-hydroxy(alkoxy)quinaldic acid chromophore, which appear to be biosynthetic components in the pathway for **1** (Figure 1 and Figure 2).

## 2. Results

### 2.1. Isolation and Structure Elucidation

The structure of verrucosamide (**1**) was initially assigned by interpretation of 1D and 2D NMR, and HRMS data. The new thiodepsipeptide was isolated as colorless crystals, and its molecular formula was determined as C_46_H_50_N_10_O_12_S_4_ based on HR-ESI-MS data ([M + Na]^+^ at *m/z* 1085.2385, calcd. for 1085.2377) in combination with NMR spectroscopic data. The IR spectrum of **1** showed absorption bands for hydroxy (3298 cm^−1^) and amide carbonyl (1647 cm^−1^) groups. ^13^C and 2D HSQC NMR spectroscopic data (Table 1 and Figures S2 and S4) revealed 23 carbons, including a thioester carbonyl at *δ*_C_ 196.8, four amide carbonyls at *δ*_C_ 172.0, 170.0, 169.2, and 168.8, nine aromatic carbons 154.2, 141.2, 135.4, 132.2, 129.3, 128.7, 127.4, 126.7, and 119.8, four nitrogen-bearing carbons at *δ*_C_ 69.8, 59.6, 55.1, and 40.8, three sulfur-bearing carbons at *δ*_C_ 29.0, 27.8, and 27.3, and two *N*-methyl carbons at *δ*_C_ 38.6 and 31.4. ^1^H and HSQC NMR spectroscopic data (Table 1 and Figures S1 and S4) showed one hydroxy proton at *δ*_H_ 11.89, two amino acid NH protons at *δ*_H_ 9.87 and 8.03, five aromatic protons at *δ*_H_ 7.75, 7.68, 7.53, 7.53, and 7.52, three nitrogen bearing methine protons at *δ*_H_ 5.33, 4.71, and 4.71, two *N*-methyl groups at *δ*_H_ 3.23 and 3.13, and four methylene groups at *δ*_H_ 4.47–2.47. However, given the molecular formula, these 23 carbons and 25 protons signals were attributed to half of the proposed molecular formula. This clearly showed that **1** is a symmetrical cyclic dimer analogous to the structures of **2** and **3**.

Interpretation of 2D COSY, HSQC, and HMBC NMR spectroscopic data for **1** led to the assignment of a 3-hydroxy-quinaldic acid unit (3HQA) and four amino acids including a cysteine (Cys), a glycine (Gly), an *N*-methylcysteine (*N*-Me-Cys), and an amino acid of unknown origin predicted, prior to ring closure, to be either an *N*-methylserine (*N*-Me-Ser) or an *N*-methyldehydroalanine (*N*-Me-dehydro-Ala), the latter of which is a prominent feature of BE-22179 (**2**, Figure 1).

**Figure 3:**
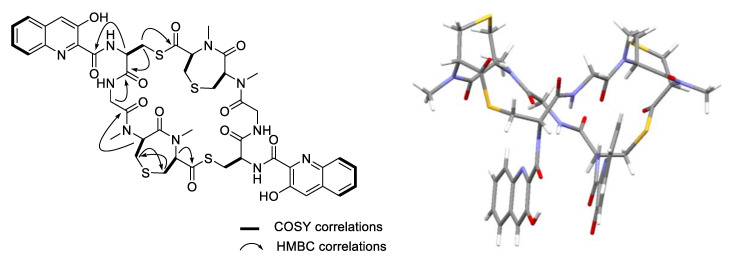
Key COSY and HMBC NMR correlations (**left**) and the X-Ray ORTEP diagram (**right**) of verrucosamide (**1**).

HMBC NMR correlations from H-2 of *N*-Me-Ser to the *N*-Me-Cys carbonyl (*δ*_C-3_ 172.0), H-4 of *N*-Me-Cys to the Gly carbonyl (*δ*_C-7_ 170.0), H_2_-8 of Gly to Cys-CO (*δ*_C-10_ 169.2), and H-11 of Cys to the 3HQA-CO (*δ*_C-13_ 168.8) revealed the half sequence of verrucosamide (**1**). Additionally, an HMBC correlation of H_2_-16 of Cys to the *N*-Me-Ser (or *N*-Me-dehydro-Ala)-carbonyl (*δ*_C-1_ 196.8) showed the attachment of sites of dimerization (Figure 3). Uniquely, *N*-Me-Ser (or *N*-Me-dehydro-Ala) and *N*-Me-Cys were connected though a sulfur atom, resulting in the formation of a rare seven-membered 1,4-thiazepane ring. The HMBC correlations between H-5 to C-6 and H-6 to C-5 confirmed this unique ring system. Thus, the planar structure of verrucosamide (**1**) was determined as the symmetrical dimer containing two 1,4-thiazepane rings.

The absolute configuration of **1** was achieved by a single-crystal X-ray diffraction (XRD) experiment. The structure assignment was possible from the anomalous signal mainly of the oxygen atoms by full-matrix least-squares refinement of the Flack parameter [17] after invariom refinement. While in an independent atom model (IAM) refinement, the value and (in brackets) standard uncertainty of the Flack parameter were 0.01 (0.22), and they could be significantly reduced to 0.08 (0.20) using an aspherical scattering model. Hence, the absolute configuration of all the chiral centers was determined as 2*R*, 4*R*, 11*S* in Figure 1.

### 2.2. Bioactivity

In order to explore the biological activity of verrucosamide (**1**), the compound was tested in the NCI 60 cell line assay. In this assay, verrucosamide showed moderate cytotoxicity and selectivity. The most susceptible cell lines were MDA-MB-468 breast carcinoma with an LD_50_ of 1.2 µM, and COLO 205 colon adenocarcinoma with an LD_50_ of 1.4 µM. By comparison, several strains of ovarian cancer and CNS cancer were far less active (see Figure S11).

### 2.3. Structures of ***4***–***6***

Along with verrucosamide, we encountered three small molecules possessing the 3-hydroxy (alkoxy) quinaldic carboxylic acid chromophore. One of these, the amide **4** was previously isolated from two *Streptomyces* sp., [18,19], but the thioesters **5** and **6** are unknown in the literature. These compounds were defined by their readily assigned spectroscopic data (Figures S7–S10).

## 3. Conclusions

Since the discovery of the bisintercalator natural product echinomycin in 1975, only about 20 functionally similar secondary metabolites have been reported. The related cyclic thiodepsipeptide compounds BE-22179 [6], thiocoraline [7,8], SW-163C [20], sandramycin [21], triostin [10], and echinomycin [11] contain a chromophore subunit that is either a 3HQA or a quinoxaline-2-carboxylic acid (QXCA) in common. In this paper we describe a new thiodepsipeptide, verrucosamide (**1**), isolated from a marine-derived *Verrucosispora* sp., strain CNX-026. The new peptide is a symmetrical dimer possessing two rare seven-membered 1,4-thiazepane rings and two 3HQA chromophores. The fundamental structure was assigned by interpretation of 1D and 2D NMR spectroscopic data. The absolute configurations of the stereogenic carbons of **1** were assigned by an X-ray diffraction experiment.

The biosynthesis of the 3HQA starting unit in verrucosamide has been well established by in vitro enzymatic experiments as being derived from *L*-tryptophan [22,23,24]. Once the corresponding starting unit has been formed in the producing microorganism, four or five amino acids are arranged sequentially by enzyme modules. Surprisingly, strain CNX-026 not only produced thiocoraline, which consisted of the 3HQA→d-Cys→GLy→l-*N*-Me-Cys→l-*N*-Me-Cys amino acid sequence, but also verrucosamide. Although verrucosamide is most closely related to BE-22179 (**2**), it is composed of an amino acid residue that appears to be preceded by _D_-*N*-Me-Ser or *N*-Me-dehydro-Ala (Figure 1). A unique component of verrucosamide are two rare seven-membered 1,4-thiazepane rings. The formation of these thiazepane rings seems linked to the homolytic cleavage of a precursor disulfide as exists in the related BE-221179. Cleavage followed by addition of an electron deficient sulfur to the exomethylene functionality of an *N*-Me-dehydro-Ala unit (also as in **2**) can readily explain the formation of these rings. Alternatively, cleavage of the disulfide could also react with a _D_-*N*-Me-Ser by dehydration, which is considerably less likely.

## 4. Materials and Methods

### 4.1. General Experimental Procedures

The optical rotations were measured using a Autopol III polarimeter (Rudolph Research, Hackettstown, NJ, USA,) equipped with a 0.1 cm cell and IR spectra were recorded on a 1600 FT-IR spectrometer (Perkin-Elmer, Waltham, MA, USA, 1H and 2D NMR spectral data were recorded at 500 MHz in acetone-*d*_6_ solution on Inova spectrometers (Varian, Palo Alto, CA, USA). ^13^C-NMR spectra were acquired at 125 MHz on a Varian Inova spectrometer. High resolution ESI-TOF mass spectra were provided by the mass spectrometry facility at the Department of Chemistry and Biochemistry at the University of California, San Diego (La Jolla, CA, USA). Data collection of diffraction intensities was performed on a SMART 6000 area detector diffractometer (Bruker, Ballerica, MA, USA) with Cu K alpha radiation generated from a 5 kW rotating anode. Flash chromatography was carried out on silica gel (230–400 mesh). Thin-layer chromatography (TLC) was performed on Polygram SIL G/UV254 (Merck, Kenilworth, NJ, USA). Rf values were measured on the same silica gel plates. Low-resolution LC/MS data were measured using a series 1100 LC/MS system (Hewlett-Packard, Palo Alto, CA, USA) with a reversed-phase C18 column (Luna, 4.6 mm × 100 mm, 5 μm, Phenomenex, Torrance, CA, USA) at a flow rate of 0.7 mL/min.

### 4.2. Strain Isolation and Identification

The marine-derived actinomycete, strain CNX-026, was isolated from a marine sediment sample collected on 19 February 2009, from the Central West Coast of Florida at latitude 27′ 44.726, longitude 82′ 34.454. The sediment was processed using ISP1 with added cyclohexamide and cultured using hydrolysate 5 g/L and yeast extract 3 g/L seawater. The 16S rRNA gene sequence of strain CNX-026 [accession number (AN): MW531553] shared 99.6% sequence identity with the *Verrucosispora andamanensis* type strain (AN: NR_109688) based on NCBI nucleotide BLAST analysis. This strain was isolated from a sponge collected from the Andaman Sea. Other closely related type strains include *Verrucosispora sediminis* MS426 (99.5%, AN: NR_116481), *Verrucosispora wenchangensis* (99.5%, AN: NR_117920) and *Verrucosispora maris* AB-18-032 (99.2%, AN: CP002638) obtained from deep-sea sediments of the South China Sea, mangrove soil from China, and deep-sea sediments from the East China Sea, respectively. Other closely related sequences deposited in the NCBI nucleotide database include *Verrucosispora* strain WMMA2109 isolated from marine sponges in Florida (100.0%, AN: KY015259) and *Verrucosispora* sp. S7-SC3 isolated from Thailand mangrove soils (99.9%, AN: KP339503).

### 4.3. Strain Cultivation

The bacterium (CNX-026) was cultured in 30 × 2.8 L Fernbach flasks each containing 1 L of a seawater based medium (10 g starch, 4 g yeast extract, 2 g peptone, 1 g CaCO_3_, 40 mg Fe_2_(SO4)_3_·4H_2_O, 100 mg KBr) and shaken at 230 rpm at 27 °C. After seven days of cultivation, sterilized XAD-16 resin (20 g/L) was added to adsorb the organic products, and the culture and resin were shaken at 215 rpm for 2 h. The resin was filtered through cheesecloth, washed with deionized water, and eluted with acetone. The acetone was removed under reduced pressure, and the resulting aqueous layer was extracted with EtOAc (3 × 500 mL). The EtOAc soluble fraction was dried in vacuo to yield 3.5 g of extract.

### 4.4. Extraction and Purification

The crude organic extract (3.5 g) was fractionated by open column chromatography on silica gel (100 g), eluted with a step gradient of CH_2_Cl_2_ and MeOH. The fraction 3 was eluted with CH_2_Cl_2_/MeOH (5:1) and it contained a mixture of both thiocoraline (**3**, 10.5 mg) and verrucosamide (**1**, 20.4 mg), which were purified by reversed-phase HPLC (Phenomenex Ultracarb C30, 250 × 100 mm, 2.5 mL/min, 5 μm, UV; 350 nm) using a gradient solvent system from 5% to 100% CH_3_CN in water (0.05% TFA) over 60 min to afford verrucosamide (**1**, 20.4 mg). Fraction 2 was eluted from the silica gel column with CH_2_Cl_2_/MeOH (10:1) and it contained a mixture of 3-hydroxy quinoline-2-carboxamide (**4**, 3.8 mg) and derivatives **5** (4.3 mg) and **6** (3.5 mg), which were purified by reversed-phase HPLC (Phenomenex Ultracarb C30, 250 × 100 mm, 2.5 mL/min, 5 μm, UV; 350 nm) using a gradient solvent system from 5% to 100% CH_3_CN in water (0.05% TFA) over 60 min.

*Verrucosamide* (**1**): colorless crystals, R_f_ 0.42 (silica plate CH_2_Cl_2_/MeOH 95:5); ${\left[\alpha \right]}_{D}^{25}$ −146.6 (c 0.12, CH_2_Cl_2_); IR (KBr) ν_max_ 3298, 2926, 1647, 1520, 1395, 1327, 1095 cm^−1^; ^1^H-NMR (500 MHz, acetone-*d*_6_) and ^13^C-NMR (125 MHz, acetone-*d*_6_) for data see Table 1; (+)-ESIMS: m/z 1062 [M + H]^+^, 1085 (+)-HR-ESI-MS m/z 1085.2385 [M + Na]^+^ (calcd. 1085.2377 for C_46_H_50_N_10_O_12_S_4_Na).

*S-Methyl 3-methoxyquinoline-2-carbothioate* (**5**): pale yellow solid, *R*_f_ 0.52 (silica plate, CH_2_Cl_2_/MeOH 90:5); IR ν_max_ cm^−1^: 2928.6, 1675, 1591, 1453, 1204, 1343, 1251, 1195; ^1^H-NMR (500 MHz, acetone-*d*_6_) and ^13^C-NMR (125 MHz, acetone-*d*_6_) for data see Table S7; (+)-ESIMS: *m/z* 234 [M + H]^+^; (+)-HR-ESI-MS *m*/*z* 234.0583 [M + H]^+^ (calcd. 234.0583, for C_12_H_12_NO_2_S).

*S-Methyl 3-hydroxyquinoline-2-carbothioate* (**6**); yellow solid, *R*_f_ 0.57 (silica plate, CH_2_Cl_2_/MeOH 95:5); IR ν_max_ cm^−1^: 2926, 1687, 1636, 1501, 1432, 1321, 1173; ^1^H-NMR (500 MHz, acetone-*d*_6_) and ^13^C NMR (125 MHz, acetone-*d*_6_) for data see Table S7; (+)-ESIMS: *m/z* 220 [M + H]^+^; (+)-HR-ESI-MS *m*/*z* 220.0426 [M + H]^+^ (calcd. 220.0427 for C_11_H_10_NO_2_S).

### 4.5. X-ray Analysis of Verrucosamide (***1***)

Verrucosamide (**1**, 3.0 mg) was dissolved in 0.5 mL of a 1:1 mixture of MeOH/acetone. The solution was sonicated to completely dissolve the material and then 0.5 mL toluene was added and the tube was placed inside a 20 mL glass vial with a lid partially closed and allowed it to sit for 10 days. Colorless crystals formed and were filtered and lightly air dried prior to X-ray analysis. See SI Tables S1–S6 for comprehensive X-ray data.

### 4.6. Bioactivity Assays

Initial cytotoxicity bioassays of the extract were conducted using the colon carcinoma cell line HCT-116. When activity was found, more advance cytotoxicity testing was performed by the National Cancer Institute using their 60 cell line panel of numerous well-defined cancer cell lines. Data showed significant cytotoxicity and a degree of specificity toward two cell lines. Verrucosamide showed LD_50_ values of 1.26 µM against MDA-MB-468 breast carcinoma and an LD_50_ of 1.4 µM against Colo-205 colon adenocarcinoma. Specific data can be found for verrucosamide (NSC-763852) in the supporting information as Figure S11.

## Figures and Tables

**Table 1:** NMR spectroscopic data for verrucosamide (**1**).

No.	1			
	*δ*_C_, mult ^a^	*δ*_H_, mult (*J* in Hz) ^b^	COSY	HMBC
OH	-	11.89, br s	-	-
1	196.8, C	-	-	-
2	69.8, CH	4.71, t (5.5)	H-6	C-1, C-3, C-6, C-14
3	172.0, C	-	-	-
4	59.6, CH	5.33, dd (10.6, 2.9)	H-5	C3, C-5, C-7, C-15
5	27.8, CH_2_	2.81, m ^c^ 2.37, m ^c^	H-4	C-3, C-4, C-6
6	29.0, CH_2_	2.90, m ^c^	H-2	C-1, C-2, C-5
7	170.0, C	-	-	-
8	40.8, CH_2_	4.47, dd (10.6, 2.9), 4.32, dd (10.6, 2.9)	H-9	C-7
9	-	8.03, br s	H-8	-
10	169.2, C	-	-	-
11	55.1, CH	4.61, m	H-16	C-10, C-16, C-13
12	-	9.87, br d (0.6)	H-11	-
13	168.8, C	-	-	-
14	38.6, CH_3_	3.14, s (N-CH3)	-	C-2, C-3
15	31.4, CH_3_	3.23, s (N-CH3)	-	C-4, C-7
16	27.3, CH_2_	4.11, d (5.4) 3.32, d (3.4)	H-11	C-1, C-10, C-11
17	135.4, C	-	-	-
18	154.2, C	-	-	-
19	119.8, CH	7.52, m ^c^		C-17, C-18, C-23, C-23a
19a	132.2, C	-	-	-
20	126.7, CH	7.75, m ^c^	H-21, H-22	C-19, C-22, C-23a
21	129.3, CH	7.68, m ^c^	H-20, H-22, H-23	C-19a, C-23
22	128.7, CH	7.53, m ^c^	H-20, H-21, H-23	C-20, C-23, C-23a
23	127.4, CH	7.53, m ^c^	H-21, H-22	C-21, C-23, C-23a
23a	141.2, C	-	-	-

^a^ Acetone-*d*_6_, 125 MHz. ^b^ Acetone-*d*_6_, 500 MHz. ^c^ Overlapping signals.

## Supplementary Materials

The following are available online at https://www.mdpi.com/1660-3397/18/11/549/s1, Table S1: Crystal data and structure refinement for verrucosamide (**1**), Table S2: Atomic coordinates (×10^4^) and equivalent isotropic displacement parameters (Å^2^x 10^3^) for verrucosamide (**1**), Table S3: Bond lengths [Å] and angles [°] for verrucosamide (**1**), Table S4: Anisotropic displacement parameters (Å^2^ x 10^3^) for verrucosamide (**1**), Table S5: Hydrogen coordinates (× 10^4^) and isotropic displacement parameters (Å^2^ x 10^3^) for verrucosamide (**1**), Table S6: Hydrogen bonds for verrucosamide (**1**) [Å and °]. Table S7: NMR spectroscopic data for compounds **5** and **6**. Figure S1: ^1^H NMR spectrum (500 MHz, acetone-*d*_6_) of verrucosamide (**1**), Figure S2: ^13^C NMR spectrum (125 MHz, acetone-*d*_6_) of verrucosamide (**1**), Figure S3: COSY spectrum (500 MHz, acetone-*d*_6_) of verrucosamide (**1**), Figure S4: HSQC spectrum (500 MHz, acetone-*d*_6_) of verrucosamide (**1**), Figure S5: HMBC spectrum (500 MHz, acetone-*d*_6_) of verrucosamide (**1**), Figure S6: ROESY NMR spectrum (500 MHz, acetone-*d*_6_) of verrucosamide (**1**), Figure S7: ^1^H NMR spectrum (500 MHz, acetone-*d*_6_) of compound **5**, Figure S8: ^13^C NMR spectrum (125 MHz, acetone-*d*_6_) of compound **5**, Figure S9: ^1^H NMR spectrum (500 MHz, acetone-*d*_6_) of compound **6**, Figure S10: ^13^C NMR spectrum (125 MHz, acetone-*d*_6_) of compound **6**, Figure S11: NCI 60 cell line cytotoxicity data for verrucosamide.
