# Supplementary material for: Verrucosamide, a Cytotoxic 1,4-Thiazepane-Containing Thiodepsipeptide from a Marine-Derived Actinomycete
Source: Mar Drugs. 2020 Nov 5;18(11):549. doi: 10.3390/md18110549 (PMC7694325; doi:10.3390/md18110549)
Supplement: Supplementary file 1 [file marinedrugs-18-00549-s001.pdf]

Supporting Information

Verrucosamide, a Cytotoxic 1,4-Thiazepane-Containing Thiodepsipeptide from a Marine-Derived Actinomycete

Vimal Nair [1*], Min Cheol Kim [1*], James A. Golen [2], Arnold L. Rheingold [2], Gabriel A. Castro [1], Paul R. Jensen [1] and William Fenical [1,3,4,*]

1. Center for Marine Biotechnology and Biomedicine, Scripps Institution of Oceanography, University of California, San Diego, La Jolla, CA 93093-0204, USA; vimal.nair16@gmail.com (V.N.); mck008@ucsd.edu (M.C.K); g8castro@ucsd.edu (G. A. C.); pjensen@ucsd.edu (P. R. J.).
2. Department of Chemistry and Biochemistry, University of California, San Diego, La Jolla, CA, 93093, jgolen@umassd.edu (J. A. G.); arheingold@ucsd.edu (A. L. R.).
3. Skaggs School of Pharmacy and Pharmaceutical Science, University of California, San Diego, La Jolla, CA, 93093, USA
4. Moores Comprehensive Cancer Center, University of California, San Diego, La Jolla, CA, 93093, USA

Correspondence: wfenical@ucsd.edu (W.F.); Tel.: +1-858-534-2133 (W.F.)

*These authors contributed equally.

Table of Contents

Table S1. Crystal data and structure refinement for verrucosamide (**1**)……………………………..3

Table S2. Atomic coordinates (x 10^4) and equivalent isotropic displacement parameters (Å^{2}x 10^3) for verrucosamide (**1**)……………………………………….………4

Table S3. Bond lengths [Å] and angles [°] for verrucosamide (**1**) ……………………………….……..8

Table S4. Anisotropic displacement parameters (Å^{2}x 10^3) for verrucosamide (**1**)……………………24

Table S5. Hydrogen coordinates (x 10^4) and isotropic displacement parameters (Å^{2}x 10^3) for verrucosamide (**1**)………………………………………………………………30

Table S6. Hydrogen bonds for verrucosamide (**1**) [Å and °]…………………………………………35

Table S7. NMR spectroscopic data for compounds **5** and **6**…………………………………..……..35

Figure S1. ^{1}H NMR spectrum (500 MHz, acetone-*d*$_6$) of verrucosamide (**1**)…………………….……..36

Figure S2. ^{13}C NMR spectrum (125 MHz, acetone-*d*$_6$) of verrucosamide (**1**)…………..………..……..37

Figure S3. COSY spectrum (500 MHz, acetone-*d*$_6$) of verrucosamide (**1**)…………..………………38

Figure S4. HSQC spectrum (500 MHz, acetone-*d*$_6$) of verrucosamide (**1**)…………….……………39

Figure S5. HMBC spectrum (500 MHz, acetone-*d*$_6$) of verrucosamide (**1**)……………………………40

Figure S6. ROESY spectrum (500 MHz, acetone-*d*$_6$) of verrucosamide (**1**)……………..…………..41

Figure S7. ^{1}H NMR spectrum (500 MHz, acetone-*d*$_6$) of compound **5**………………….……………42

Figure S8. ^{13}C NMR spectrum (500 MHz, acetone-*d*$_6$) of compound **5**………………….…………43

Figure S9. ^{1}H NMR spectrum (500 MHz, acetone-*d*$_6$) of compound **6**………………………………..44

Figure S10. ^{13}C NMR spectrum (500 MHz, acetone-*d*$_6$) of compound **6**……………………..………45

Figure S11. NCI 60 cell line cytotoxicity results for verrucosamide (NSC S763852)…………………46

Table 1. Crystal data and structure refinement for verrucosamide (**1**).

Identification code	26F3_3
Empirical formula	C114 H130 N20 O26 S8
	(2 verrucosamide, 3 toluene, 1 MeOH and 1 H_2O)
Formula weight	1226.43
Temperature	120(2) K
Wavelength	1.54178 Å
Crystal system	Orthorhombic
Space group	P2(1)2(1)2(1)
Unit cell dimensions	a = 16.2112(5) Å α= 90°.
	b = 18.3755(6) Å β= 90°.
	c = 39.4613(16) Å γ = 90°.
Volume	11755.1(7) $Å^3$
Z	8
Density (calculated)	1.386 Mg/m^3
Absorption coefficient	2.091 mm^{-1}
F(000)	5160
Crystal size	0.11 x 0.02 x 0.01 mm^3
Crystal color, habit	Colorless Needle
Theta range for data collection	2.65 to 60.11°.
Index ranges	-18<=h<=18, -20<=k<=20, -44<=l<=44
Reflections collected	16581
Independent reflections	16581 [R(int) = 0.0781]
Completeness to theta = 60.00°	98.1 %
Absorption correction	Semi-empirical from equivalents
Max. and min. transmission	0.9794 and 0.8026
Refinement method	Full-matrix least-squares on F^2
Data / restraints / parameters	16581 / 60 / 1457
Goodness-of-fit on F^2	1.040
Final R indices [I>2sigma(I)]	R1 = 0.0795, wR2 = 0.2059
R indices (all data)	R1 = 0.1096, wR2 = 0.2232
Absolute structure parameter	0.01(2)
Extinction coefficient	not measured
Largest diff. peak and hole	0.564 and -0.538 e.$Å^{-3}$

Table 2. Atomic coordinates (x 10^4) and equivalent isotropic displacement parameters ($Å^2$x 10^3) for verrucosamide (**1**). U(eq) is defined as one third of the trace of the orthogonalized U^{ij} tensor.

	x	y	z	U(eq)
S(1)	6589(1)	14831(1)	9649(1)	54(1)
S(2)	6670(2)	14086(1)	8554(1)	66(1)
S(3)	11776(1)	16918(1)	9176(1)	50(1)
S(4)	11662(1)	15795(1)	8131(1)	59(1)
O(1)	8579(4)	16874(3)	10785(1)	60(1)
O(2)	7644(3)	16899(3)	10241(1)	58(1)
O(3)	5468(3)	15027(3)	9171(2)	72(2)
O(4)	7846(3)	13051(3)	9554(2)	67(2)
O(5)	8677(3)	14925(3)	9375(1)	51(1)
O(6)	11620(3)	14789(3)	9416(2)	70(2)
O(7)	12767(3)	15981(3)	8859(2)	65(2)
O(8)	10334(3)	17903(3)	8580(2)	75(2)
O(9)	9684(3)	16423(3)	9044(1)	49(1)
O(10)	6791(3)	16230(3)	9041(1)	63(1)
O(11)	10956(3)	16723(3)	10231(1)	52(1)
O(12)	10252(4)	17753(3)	10540(1)	58(1)
N(1)	9042(4)	15439(3)	10179(2)	55(2)
N(2)	7831(4)	16056(3)	9837(2)	52(2)
N(3)	6573(4)	13358(4)	9379(2)	61(2)
N(4)	8700(4)	13826(3)	9112(2)	51(2)
N(5)	10280(4)	15073(3)	9420(2)	50(2)
N(6)	10580(3)	16423(3)	9698(2)	45(1)
N(7)	11647(4)	17516(4)	8512(2)	58(2)
N(8)	9564(4)	16673(4)	8491(2)	55(2)
N(9)	8094(3)	16502(3)	9175(2)	49(1)

N(10)	9527(4)	17560(3)	9677(2)	52(2)
C(1)	9704(5)	15126(4)	10328(2)	53(2)
C(2)	10045(6)	14508(5)	10177(2)	66(2)
C(3)	10770(6)	14210(5)	10298(3)	75(3)
C(4)	11151(6)	14536(5)	10578(3)	76(3)
C(5)	10803(5)	15097(5)	10744(2)	64(2)
C(6)	10048(5)	15420(4)	10626(2)	57(2)
C(7)	9651(5)	15988(4)	10785(2)	57(2)
C(8)	8964(5)	16300(4)	10634(2)	52(2)
C(9)	8705(4)	16018(3)	10319(2)	43(2)
C(10)	8005(5)	16357(4)	10132(2)	47(2)
C(11)	7116(5)	16262(4)	9628(2)	53(2)
C(12)	6370(5)	15785(4)	9701(2)	59(2)
C(13)	5951(5)	14602(5)	9300(2)	59(2)
C(14)	5958(5)	13816(4)	9203(2)	60(2)
C(15)	5894(5)	13722(5)	8817(3)	68(2)
C(16)	7542(5)	13600(4)	8719(2)	56(2)
C(17)	7807(5)	13841(4)	9070(2)	59(2)
C(18)	7404(5)	13382(4)	9359(2)	55(2)
C(19)	6249(6)	12891(5)	9646(3)	82(3)
C(20)	9181(5)	13211(4)	8986(2)	61(2)
C(21)	9055(5)	14401(4)	9267(2)	51(2)
C(22)	9999(5)	14379(4)	9299(2)	51(2)
C(23)	11078(4)	15222(4)	9473(2)	46(2)
C(24)	11279(4)	15925(4)	9662(2)	50(2)
C(25)	12032(4)	16309(4)	9520(2)	46(2)
C(26)	12331(4)	16512(4)	8833(2)	47(2)
C(27)	12294(4)	16955(4)	8514(2)	56(2)
C(28)	12398(5)	16508(5)	8195(2)	61(2)
C(29)	10708(4)	16324(5)	8112(2)	58(2)

C(30)	10482(4)	16644(4)	8452(2)	51(2)
C(31)	10809(5)	17393(5)	8526(2)	58(2)
C(32)	11932(6)	18261(5)	8573(3)	77(3)
C(33)	9103(5)	16922(5)	8188(2)	64(2)
C(34)	9257(4)	16564(4)	8800(2)	48(2)
C(35)	8327(5)	16612(4)	8828(2)	53(2)
C(36)	7319(4)	16322(4)	9256(2)	46(2)
C(37)	10532(4)	16827(4)	9976(2)	44(2)
C(38)	9916(4)	17453(3)	9968(2)	40(2)
C(39)	9819(4)	17892(4)	10255(2)	42(2)
C(40)	9313(5)	18474(4)	10237(2)	53(2)
C(41)	8902(4)	18635(4)	9933(2)	50(2)
C(42)	9033(5)	18157(4)	9650(2)	50(2)
C(43)	8634(7)	18315(5)	9340(3)	77(3)
C(44)	8151(7)	18899(5)	9301(3)	91(4)
C(45)	8031(5)	19365(5)	9581(3)	79(3)
C(46)	8382(5)	19252(4)	9887(3)	64(2)
S(1')	4904(1)	13475(1)	6857(1)	49(1)
S(2')	5305(1)	10933(1)	6565(1)	54(1)
S(3')	3409(1)	11763(1)	8978(1)	66(1)
S(4')	3865(1)	14076(1)	9477(1)	70(1)
O(1')	9058(4)	12938(5)	7214(2)	93(2)
O(2')	7495(3)	13061(4)	7189(2)	72(2)
O(3')	6082(3)	12606(3)	6664(2)	77(2)
O(4')	2836(3)	12269(3)	6792(1)	53(1)
O(5')	4400(4)	12076(3)	7556(1)	60(1)
O(6')	4400(6)	10472(3)	8238(2)	99(3)
O(7')	4247(4)	12315(4)	9491(2)	72(2)
O(8')	1981(3)	13322(3)	8629(2)	72(2)
O(9')	4332(3)	13047(3)	8356(1)	52(1)

O(10')	5502(3)	14536(3)	7768(2)	74(2)
O(11')	6296(4)	11895(3)	8897(2)	67(2)
O(12')	7854(5)	11727(4)	9010(2)	94(2)
N(1')	8221(4)	14152(3)	7854(2)	54(2)
N(2')	6795(4)	13786(3)	7537(2)	50(2)
N(3')	4664(4)	13579(3)	7720(2)	55(2)
N(4')	3422(4)	13927(3)	8482(2)	54(2)
N(5')	2312(4)	12946(4)	9156(2)	67(2)
N(6')	5608(5)	11048(4)	8597(2)	61(2)
N(7')	3847(5)	11571(4)	8151(2)	60(2)
N(8')	3578(4)	11378(3)	7235(2)	48(2)
N(9')	3947(4)	12444(3)	6456(2)	45(1)
N(10')	6993(4)	10312(3)	8486(2)	61(2)
C(1')	8945(5)	14314(5)	8028(2)	54(2)
C(2')	8900(5)	14774(4)	8306(2)	58(2)
C(3')	9567(5)	14906(4)	8504(2)	59(2)
C(4')	10323(6)	14572(5)	8418(2)	67(2)
C(5')	10394(5)	14144(6)	8149(2)	72(3)
C(6')	9700(5)	13986(5)	7940(2)	61(2)
C(7')	9739(5)	13540(6)	7661(2)	75(3)
C(8')	9020(5)	13390(6)	7487(3)	71(3)
C(9')	8274(5)	13701(5)	7595(2)	55(2)
C(10')	7478(5)	13491(4)	7426(2)	50(2)
C(11')	5969(4)	13572(4)	7419(2)	47(2)
C(12')	5819(5)	13861(4)	7059(2)	52(2)
C(13')	5382(5)	12736(4)	6627(2)	51(2)
C(14')	4813(4)	12372(4)	6370(2)	43(2)
C(15')	5125(4)	11623(4)	6248(2)	47(2)
C(16')	4262(5)	10882(4)	6728(2)	50(2)
C(17')	4035(4)	11570(4)	6930(2)	44(2)

C(18')	3553(5)	12130(4)	6721(2)	44(2)
C(19')	3456(5)	12927(4)	6232(2)	57(2)
C(20')	2889(5)	10867(4)	7203(2)	56(2)
C(21')	3825(5)	11643(4)	7532(2)	51(2)
C(22')	3397(6)	11363(5)	7846(2)	63(2)
C(23')	4333(6)	11113(5)	8312(2)	68(2)
C(24')	4829(6)	11424(4)	8605(2)	64(2)
C(25')	4380(6)	11253(5)	8949(2)	67(2)
C(26')	3619(6)	12328(5)	9333(2)	61(2)
C(27')	2886(5)	12817(5)	9436(2)	66(2)
C(28')	3094(6)	13473(5)	9646(3)	73(3)
C(29')	3401(5)	14333(4)	9077(2)	61(2)
C(30')	3314(5)	13684(4)	8831(2)	52(2)
C(31')	2469(5)	13300(4)	8866(3)	61(2)
C(32')	1472(6)	12653(6)	9204(3)	89(3)
C(33')	2913(5)	14550(5)	8367(2)	64(2)
C(34')	3942(4)	13583(4)	8271(2)	48(2)
C(35')	4023(5)	13895(4)	7927(2)	61(2)
C(36')	5358(5)	13925(4)	7658(2)	49(2)
C(37')	6285(5)	11324(4)	8728(2)	57(2)
C(38')	7052(5)	10897(4)	8673(2)	56(2)
C(39')	7828(6)	11137(5)	8813(3)	73(3)
C(40')	8512(6)	10743(6)	8744(3)	79(3)
C(41')	8464(6)	10135(5)	8536(3)	71(2)
C(42')	7686(5)	9920(5)	8418(2)	64(2)
C(43')	7611(7)	9271(5)	8214(3)	82(3)
C(44')	8286(8)	8873(5)	8138(3)	97(4)
C(45')	9071(8)	9100(7)	8261(4)	98(4)
C(46')	9178(8)	9696(7)	8454(3)	96(3)
O(1S)	9513(5)	12840(4)	9767(2)	88(2)

O(2S)	7504(9)	17836(9)	7008(5)	222(7)
C(1SM)	7942(15)	17790(13)	7424(7)	222(7)
C(1S)	13861(10)	15677(7)	7439(3)	126(5)
C(2S)	13312(9)	16195(5)	7267(2)	87(3)
C(3S)	12458(8)	16118(6)	7257(3)	91(3)
C(4S)	11941(8)	16594(6)	7102(3)	90(3)
C(5S)	12276(8)	17183(6)	6952(3)	87(3)
C(6S)	13116(8)	17317(5)	6956(2)	82(3)
C(7S)	13643(7)	16834(6)	7111(2)	80(3)
C(8S)	4864(10)	13996(10)	10404(4)	145(2)
C(9S)	5497(12)	14524(10)	10509(5)	145(2)
C(10S)	5344(12)	15213(10)	10653(4)	145(2)
C(11S)	5863(11)	15727(11)	10759(4)	145(2)
C(12S)	6722(12)	15461(10)	10737(4)	145(2)
C(13S)	6989(11)	14811(10)	10591(4)	145(2)
C(14S)	6391(11)	14304(11)	10485(5)	145(2)
C(15S)	9618(14)	17081(12)	6708(6)	200(4)
C(16S)	9215(15)	16404(12)	6809(6)	200(4)
C(17S)	9431(14)	16065(13)	7123(6)	200(4)
C(18S)	9053(15)	15432(13)	7183(6)	200(4)
C(19S)	8522(14)	15166(12)	6954(6)	200(4)
C(20S)	8236(15)	15424(13)	6647(6)	200(4)
C(21S)	8646(14)	16077(12)	6573(6)	200(4)

Table 3. Bond lengths [Å] and angles [°] for verrucosamide (**1**).

S(1)-C(13)	1.772(9)	N(3)-C(14)	1.480(10)
S(1)-C(12)	1.801(8)	N(4)-C(21)	1.351(10)
S(2)-C(15)	1.762(10)	N(4)-C(17)	1.457(10)
S(2)-C(16)	1.794(8)	N(4)-C(20)	1.461(10)
S(3)-C(26)	1.787(8)	N(5)-C(23)	1.338(9)
S(3)-C(25)	1.807(8)	N(5)-C(22)	1.436(9)
S(4)-C(28)	1.790(9)	N(5)-H(5)	0.8800
S(4)-C(29)	1.828(7)	N(6)-C(37)	1.327(9)
O(1)-C(8)	1.362(10)	N(6)-C(24)	1.463(9)
O(1)-H(1)	0.8400	N(6)-H(6)	0.8800
O(2)-C(10)	1.233(9)	N(7)-C(31)	1.379(10)
O(3)-C(13)	1.218(9)	N(7)-C(32)	1.465(11)
O(4)-C(18)	1.216(10)	N(7)-C(27)	1.471(10)
O(5)-C(21)	1.218(9)	N(8)-C(34)	1.335(10)
O(6)-C(23)	1.206(9)	N(8)-C(33)	1.484(10)
O(7)-C(26)	1.209(9)	N(8)-C(30)	1.497(9)
O(8)-C(31)	1.231(10)	N(9)-C(36)	1.338(10)
O(9)-C(34)	1.214(9)	N(9)-C(35)	1.434(10)
O(10)-C(36)	1.217(9)	N(9)-H(9)	0.8800
O(11)-C(37)	1.232(9)	N(10)-C(38)	1.326(9)
O(12)-C(39)	1.350(9)	N(10)-C(42)	1.363(10)
O(12)-H(12)	0.8400	C(1)-C(2)	1.398(12)
N(1)-C(9)	1.319(9)	C(1)-C(6)	1.407(11)
N(1)-C(1)	1.352(10)	C(2)-C(3)	1.383(13)
N(2)-C(10)	1.317(10)	C(2)-H(2A)	0.9500
N(2)-C(11)	1.473(10)	C(3)-C(4)	1.400(15)
N(2)-H(2)	0.8800	C(3)-H(3)	0.9500
N(3)-C(18)	1.351(11)	C(4)-C(5)	1.344(14)
N(3)-C(19)	1.456(12)	C(4)-H(4)	0.9500

C(5)-C(6)	1.439(11)	C(22)-H(22D)	0.9900
C(5)-H(5A)	0.9500	C(23)-C(24)	1.528(10)
C(6)-C(7)	1.377(12)	C(24)-C(25)	1.517(10)
C(7)-C(8)	1.386(11)	C(24)-H(24)	1.0000
C(7)-H(7)	0.9500	C(25)-H(25C)	0.9900
C(8)-C(9)	1.410(10)	C(25)-H(25D)	0.9900
C(9)-C(10)	1.490(10)	C(26)-C(27)	1.501(11)
C(11)-C(36)	1.510(11)	C(27)-C(28)	1.513(12)
C(11)-C(12)	1.521(12)	C(27)-H(27)	1.0000
C(11)-H(11)	1.0000	C(28)-H(28C)	0.9900
C(12)-H(12C)	0.9900	C(28)-H(28D)	0.9900
C(12)-H(12D)	0.9900	C(29)-C(30)	1.509(11)
C(13)-C(14)	1.494(12)	C(29)-H(29C)	0.9900
C(14)-C(15)	1.534(13)	C(29)-H(29D)	0.9900
C(14)-H(14)	1.0000	C(30)-C(31)	1.504(12)
C(15)-H(15C)	0.9900	C(30)-H(30)	1.0000
C(15)-H(15D)	0.9900	C(32)-H(32D)	0.9800
C(16)-C(17)	1.517(12)	C(32)-H(32E)	0.9800
C(16)-H(16C)	0.9900	C(32)-H(32F)	0.9800
C(16)-H(16D)	0.9900	C(33)-H(33D)	0.9800
C(17)-C(18)	1.561(12)	C(33)-H(33E)	0.9800
C(17)-H(17)	1.0000	C(33)-H(33F)	0.9800
C(19)-H(19D)	0.9800	C(34)-C(35)	1.514(10)
C(19)-H(19E)	0.9800	C(35)-H(35C)	0.9900
C(19)-H(19F)	0.9800	C(35)-H(35D)	0.9900
C(20)-H(20D)	0.9800	C(37)-C(38)	1.523(10)
C(20)-H(20E)	0.9800	C(38)-C(39)	1.400(10)
C(20)-H(20F)	0.9800	C(39)-C(40)	1.349(11)
C(21)-C(22)	1.536(11)	C(40)-C(41)	1.406(11)
C(22)-H(22C)	0.9900	C(40)-H(40)	0.9500

C(41)-C(46)	1.424(11)	O(12')-C(39')	1.333(12)
C(41)-C(42)	1.434(11)	O(12')-H(12')	0.8400
C(42)-C(43)	1.416(12)	N(1')-C(9')	1.318(10)
C(43)-C(44)	1.337(14)	N(1')-C(1')	1.393(10)
C(43)-H(43)	0.9500	N(2')-C(10')	1.308(10)
C(44)-C(45)	1.413(16)	N(2')-C(11')	1.471(9)
C(44)-H(44)	0.9500	N(2')-H(2')	0.8800
C(45)-C(46)	1.353(14)	N(3')-C(36')	1.316(10)
C(45)-H(45)	0.9500	N(3')-C(35')	1.445(11)
C(46)-H(46)	0.9500	N(3')-H(3')	0.8800
S(1')-C(13')	1.807(8)	N(4')-C(34')	1.343(10)
S(1')-C(12')	1.827(7)	N(4')-C(30')	1.460(10)
S(2')-C(15')	1.802(7)	N(4')-C(33')	1.481(10)
S(2')-C(16')	1.810(8)	N(5')-C(31')	1.341(12)
S(3')-C(26')	1.776(10)	N(5')-C(27')	1.462(12)
S(3')-C(25')	1.835(9)	N(5')-C(32')	1.476(12)
S(4')-C(28')	1.799(10)	N(6')-C(37')	1.316(11)
S(4')-C(29')	1.811(9)	N(6')-C(24')	1.439(11)
O(1')-C(8')	1.362(11)	N(6')-H(6')	0.8800
O(1')-H(1')	0.8400	N(7')-C(23')	1.317(11)
O(2')-C(10')	1.226(9)	N(7')-C(22')	1.458(10)
O(3')-C(13')	1.169(9)	N(7')-H(7')	0.8800
O(4')-C(18')	1.223(9)	N(8')-C(21')	1.331(10)
O(5')-C(21')	1.228(9)	N(8')-C(17')	1.456(10)
O(6')-C(23')	1.219(11)	N(8')-C(20')	1.465(9)
O(7')-C(26')	1.195(11)	N(9')-C(18')	1.354(9)
O(8')-C(31')	1.226(11)	N(9')-C(14')	1.450(9)
O(9')-C(34')	1.218(9)	N(9')-C(19')	1.485(9)
O(10')-C(36')	1.225(9)	N(10')-C(38')	1.307(10)
O(11')-C(37')	1.244(9)	N(10')-C(42')	1.361(11)

C(1')-C(2')	1.387(11)	C(19')-H(19A)	0.9800
C(1')-C(6')	1.408(12)	C(19')-H(19B)	0.9800
C(2')-C(3')	1.357(12)	C(19')-H(19C)	0.9800
C(2')-H(2'A)	0.9500	C(20')-H(20A)	0.9800
C(3')-C(4')	1.411(13)	C(20')-H(20B)	0.9800
C(3')-H(3'A)	0.9500	C(20')-H(20C)	0.9800
C(4')-C(5')	1.325(13)	C(21')-C(22')	1.512(11)
C(4')-H(4')	0.9500	C(22')-H(22A)	0.9900
C(5')-C(6')	1.426(12)	C(22')-H(22B)	0.9900
C(5')-H(5')	0.9500	C(23')-C(24')	1.519(13)
C(6')-C(7')	1.372(13)	C(24')-C(25')	1.573(13)
C(7')-C(8')	1.380(13)	C(24')-H(24')	1.0000
C(7')-H(7'A)	0.9500	C(25')-H(25A)	0.9900
C(8')-C(9')	1.403(12)	C(25')-H(25B)	0.9900
C(9')-C(10')	1.502(11)	C(26')-C(27')	1.543(12)
C(11')-C(36')	1.513(11)	C(27')-C(28')	1.501(13)
C(11')-C(12')	1.537(11)	C(27')-H(27')	1.0000
C(11')-H(11')	1.0000	C(28')-H(28A)	0.9900
C(12')-H(12A)	0.9900	C(28')-H(28B)	0.9900
C(12')-H(12B)	0.9900	C(29')-C(30')	1.545(10)
C(13')-C(14')	1.524(10)	C(29')-H(29A)	0.9900
C(14')-C(15')	1.544(10)	C(29')-H(29B)	0.9900
C(14')-H(14')	1.0000	C(30')-C(31')	1.548(11)
C(15')-H(15A)	0.9900	C(30')-H(30')	1.0000
C(15')-H(15B)	0.9900	C(32')-H(32A)	0.9800
C(16')-C(17')	1.540(10)	C(32')-H(32B)	0.9800
C(16')-H(16A)	0.9900	C(32')-H(32C)	0.9800
C(16')-H(16B)	0.9900	C(33')-H(33A)	0.9800
C(17')-C(18')	1.534(10)	C(33')-H(33B)	0.9800
C(17')-H(17')	1.0000	C(33')-H(33C)	0.9800

C(34')-C(35')	1.479(12)	C(2S)-C(7S)	1.431(14)
C(35')-H(35A)	0.9900	C(3S)-C(4S)	1.356(15)
C(35')-H(35B)	0.9900	C(3S)-H(3S)	0.9500
C(37')-C(38')	1.486(12)	C(4S)-C(5S)	1.348(15)
C(38')-C(39')	1.443(13)	C(4S)-H(4S)	0.9500
C(39')-C(40')	1.353(14)	C(5S)-C(6S)	1.383(15)
C(40')-C(41')	1.388(14)	C(5S)-H(5S)	0.9500
C(40')-H(40')	0.9500	C(6S)-C(7S)	1.374(14)
C(41')-C(42')	1.402(13)	C(6S)-H(6S)	0.9500
C(41')-C(46')	1.447(15)	C(7S)-H(7S)	0.9500
C(42')-C(43')	1.444(14)	C(8S)-C(9S)	1.47(2)
C(43')-C(44')	1.349(15)	C(8S)-H(8SA)	0.9800
C(43')-H(43')	0.9500	C(8S)-H(8SB)	0.9800
C(44')-C(45')	1.425(17)	C(8S)-H(8SC)	0.9800
C(44')-H(44')	0.9500	C(9S)-C(10S)	1.41(2)
C(45')-C(46')	1.345(17)	C(9S)-C(14S)	1.51(2)
C(45')-H(45')	0.9500	C(10S)-C(11S)	1.33(2)
C(46')-H(46')	0.9500	C(10S)-H(10S)	0.9500
O(1S)-H(1SD)	0.91(2)	C(11S)-C(12S)	1.48(2)
O(1S)-H(1SE)	0.91(2)	C(11S)-H(11S)	0.9500
O(2S)-C(1SM)	1.79(3)	C(12S)-C(13S)	1.40(2)
O(2S)-H(2S)	0.8400	C(12S)-H(12S)	0.9500
C(1SM)-H(1SA)	0.9800	C(13S)-C(14S)	1.41(2)
C(1SM)-H(1SB)	0.9800	C(13S)-H(13S)	0.9500
C(1SM)-H(1SC)	0.9800	C(14S)-H(14S)	0.9500
C(1S)-C(2S)	1.470(15)	C(15S)-C(16S)	1.461(19)
C(1S)-H(1SF)	0.9800	C(15S)-H(15E)	0.9800
C(1S)-H(1SG)	0.9800	C(15S)-H(15F)	0.9800
C(1S)-H(1SH)	0.9800	C(15S)-H(15G)	0.9800
C(2S)-C(3S)	1.393(16)	C(16S)-C(17S)	1.43(2)

C(16S)-C(21S)	1.442(19)
C(17S)-C(18S)	1.34(2)
C(17S)-H(17S)	0.9500
C(18S)-C(19S)	1.340(19)
C(18S)-H(18S)	0.9500
C(19S)-C(20S)	1.382(19)
C(19S)-H(19S)	0.9500
C(20S)-C(21S)	1.403(19)
C(20S)-H(20S)	0.9500
C(21S)-H(21S)	0.9500
C(13)-S(1)-C(12)	101.8(4)
C(15)-S(2)-C(16)	99.3(4)
C(26)-S(3)-C(25)	101.2(3)
C(28)-S(4)-C(29)	100.4(4)
C(8)-O(1)-H(1)	109.5
C(39)-O(12)-H(12)	109.5
C(9)-N(1)-C(1)	119.2(7)
C(10)-N(2)-C(11)	123.7(6)
C(10)-N(2)-H(2)	118.2
C(11)-N(2)-H(2)	118.2
C(18)-N(3)-C(19)	115.0(7)
C(18)-N(3)-C(14)	128.7(7)
C(19)-N(3)-C(14)	115.7(7)
C(21)-N(4)-C(17)	117.4(6)
C(21)-N(4)-C(20)	122.2(7)
C(17)-N(4)-C(20)	120.4(6)
C(23)-N(5)-C(22)	122.6(6)
C(23)-N(5)-H(5)	118.7
C(22)-N(5)-H(5)	118.7
C(37)-N(6)-C(24)	118.4(6)
C(37)-N(6)-H(6)	120.8
C(24)-N(6)-H(6)	120.8
C(31)-N(7)-C(32)	117.2(7)
C(31)-N(7)-C(27)	126.0(7)
C(32)-N(7)-C(27)	115.4(6)
C(34)-N(8)-C(33)	126.6(6)
C(34)-N(8)-C(30)	117.5(6)
C(33)-N(8)-C(30)	115.4(6)
C(36)-N(9)-C(35)	120.7(6)
C(36)-N(9)-H(9)	119.7
C(35)-N(9)-H(9)	119.7
C(38)-N(10)-C(42)	117.8(6)
N(1)-C(1)-C(2)	118.1(7)
N(1)-C(1)-C(6)	121.0(7)
C(2)-C(1)-C(6)	120.8(8)
C(3)-C(2)-C(1)	120.6(9)
C(3)-C(2)-H(2A)	119.7
C(1)-C(2)-H(2A)	119.7
C(2)-C(3)-C(4)	118.7(9)
C(2)-C(3)-H(3)	120.7
C(4)-C(3)-H(3)	120.7
C(5)-C(4)-C(3)	121.7(8)
C(5)-C(4)-H(4)	119.1
C(3)-C(4)-H(4)	119.1
C(4)-C(5)-C(6)	121.1(9)
C(4)-C(5)-H(5A)	119.4
C(6)-C(5)-H(5A)	119.4
C(7)-C(6)-C(1)	119.0(7)
C(7)-C(6)-C(5)	124.3(8)

C(1)-C(6)-C(5)	116.6(8)	C(13)-C(14)-C(15)	111.2(7)
C(6)-C(7)-C(8)	119.6(8)	N(3)-C(14)-H(14)	103.7
C(6)-C(7)-H(7)	120.2	C(13)-C(14)-H(14)	103.7
C(8)-C(7)-H(7)	120.2	C(15)-C(14)-H(14)	103.7
O(1)-C(8)-C(7)	120.0(7)	C(14)-C(15)-S(2)	119.6(6)
O(1)-C(8)-C(9)	122.2(7)	C(14)-C(15)-H(15C)	107.4
C(7)-C(8)-C(9)	117.7(7)	S(2)-C(15)-H(15C)	107.4
N(1)-C(9)-C(8)	122.9(7)	C(14)-C(15)-H(15D)	107.4
N(1)-C(9)-C(10)	116.4(6)	S(2)-C(15)-H(15D)	107.4
C(8)-C(9)-C(10)	120.8(7)	H(15C)-C(15)-H(15D)	107.0
O(2)-C(10)-N(2)	123.1(7)	C(17)-C(16)-S(2)	114.1(6)
O(2)-C(10)-C(9)	121.6(7)	C(17)-C(16)-H(16C)	108.7
N(2)-C(10)-C(9)	115.2(7)	S(2)-C(16)-H(16C)	108.7
N(2)-C(11)-C(36)	113.1(6)	C(17)-C(16)-H(16D)	108.7
N(2)-C(11)-C(12)	111.8(6)	S(2)-C(16)-H(16D)	108.7
C(36)-C(11)-C(12)	113.5(7)	H(16C)-C(16)-H(16D)	107.6
N(2)-C(11)-H(11)	105.9	N(4)-C(17)-C(16)	112.2(7)
C(36)-C(11)-H(11)	105.9	N(4)-C(17)-C(18)	108.9(7)
C(12)-C(11)-H(11)	105.9	C(16)-C(17)-C(18)	113.0(6)
C(11)-C(12)-S(1)	112.5(5)	N(4)-C(17)-H(17)	107.5
C(11)-C(12)-H(12C)	109.1	C(16)-C(17)-H(17)	107.5
S(1)-C(12)-H(12C)	109.1	C(18)-C(17)-H(17)	107.5
C(11)-C(12)-H(12D)	109.1	O(4)-C(18)-N(3)	122.2(8)
S(1)-C(12)-H(12D)	109.1	O(4)-C(18)-C(17)	119.2(7)
H(12C)-C(12)-H(12D)	107.8	N(3)-C(18)-C(17)	118.6(7)
O(3)-C(13)-C(14)	121.1(8)	N(3)-C(19)-H(19D)	109.5
O(3)-C(13)-S(1)	123.2(7)	N(3)-C(19)-H(19E)	109.5
C(14)-C(13)-S(1)	115.1(6)	H(19D)-C(19)-H(19E)	109.5
N(3)-C(14)-C(13)	115.7(7)	N(3)-C(19)-H(19F)	109.5
N(3)-C(14)-C(15)	116.6(8)	H(19D)-C(19)-H(19F)	109.5

H(19E)-C(19)-H(19F)	109.5	H(25C)-C(25)-H(25D)	107.9
N(4)-C(20)-H(20D)	109.5	O(7)-C(26)-C(27)	122.1(7)
N(4)-C(20)-H(20E)	109.5	O(7)-C(26)-S(3)	124.6(6)
H(20D)-C(20)-H(20E)	109.5	C(27)-C(26)-S(3)	112.9(5)
N(4)-C(20)-H(20F)	109.5	N(7)-C(27)-C(26)	114.4(7)
H(20D)-C(20)-H(20F)	109.5	N(7)-C(27)-C(28)	117.0(7)
H(20E)-C(20)-H(20F)	109.5	C(26)-C(27)-C(28)	113.5(6)
O(5)-C(21)-N(4)	124.2(7)	N(7)-C(27)-H(27)	103.1
O(5)-C(21)-C(22)	119.6(7)	C(26)-C(27)-H(27)	103.1
N(4)-C(21)-C(22)	116.2(7)	C(28)-C(27)-H(27)	103.1
N(5)-C(22)-C(21)	108.7(6)	C(27)-C(28)-S(4)	116.2(6)
N(5)-C(22)-H(22C)	110.0	C(27)-C(28)-H(28C)	108.2
C(21)-C(22)-H(22C)	110.0	S(4)-C(28)-H(28C)	108.2
N(5)-C(22)-H(22D)	110.0	C(27)-C(28)-H(28D)	108.2
C(21)-C(22)-H(22D)	110.0	S(4)-C(28)-H(28D)	108.2
H(22C)-C(22)-H(22D)	108.3	H(28C)-C(28)-H(28D)	107.4
O(6)-C(23)-N(5)	122.8(7)	C(30)-C(29)-S(4)	112.1(6)
O(6)-C(23)-C(24)	119.5(6)	C(30)-C(29)-H(29C)	109.2
N(5)-C(23)-C(24)	117.1(6)	S(4)-C(29)-H(29C)	109.2
N(6)-C(24)-C(25)	111.7(6)	C(30)-C(29)-H(29D)	109.2
N(6)-C(24)-C(23)	114.2(6)	S(4)-C(29)-H(29D)	109.2
C(25)-C(24)-C(23)	112.6(6)	H(29C)-C(29)-H(29D)	107.9
N(6)-C(24)-H(24)	105.8	N(8)-C(30)-C(31)	107.3(6)
C(25)-C(24)-H(24)	105.8	N(8)-C(30)-C(29)	110.3(6)
C(23)-C(24)-H(24)	105.8	C(31)-C(30)-C(29)	116.3(7)
C(24)-C(25)-S(3)	112.4(5)	N(8)-C(30)-H(30)	107.5
C(24)-C(25)-H(25C)	109.1	C(31)-C(30)-H(30)	107.5
S(3)-C(25)-H(25C)	109.1	C(29)-C(30)-H(30)	107.5
C(24)-C(25)-H(25D)	109.1	O(8)-C(31)-N(7)	119.9(8)
S(3)-C(25)-H(25D)	109.1	O(8)-C(31)-C(30)	120.7(7)

N(7)-C(31)-C(30)	119.3(7)
N(7)-C(32)-H(32D)	109.5
N(7)-C(32)-H(32E)	109.5
H(32D)-C(32)-H(32E)	109.5
N(7)-C(32)-H(32F)	109.5
H(32D)-C(32)-H(32F)	109.5
H(32E)-C(32)-H(32F)	109.5
N(8)-C(33)-H(33D)	109.5
N(8)-C(33)-H(33E)	109.5
H(33D)-C(33)-H(33E)	109.5
N(8)-C(33)-H(33F)	109.5
H(33D)-C(33)-H(33F)	109.5
H(33E)-C(33)-H(33F)	109.5
O(9)-C(34)-N(8)	123.0(6)
O(9)-C(34)-C(35)	121.6(7)
N(8)-C(34)-C(35)	115.4(6)
N(9)-C(35)-C(34)	108.8(6)
N(9)-C(35)-H(35C)	109.9
C(34)-C(35)-H(35C)	109.9
N(9)-C(35)-H(35D)	109.9
C(34)-C(35)-H(35D)	109.9
H(35C)-C(35)-H(35D)	108.3
O(10)-C(36)-N(9)	121.9(7)
O(10)-C(36)-C(11)	121.1(7)
N(9)-C(36)-C(11)	117.0(6)
O(11)-C(37)-N(6)	123.7(7)
O(11)-C(37)-C(38)	120.0(6)
N(6)-C(37)-C(38)	116.4(6)
N(10)-C(38)-C(39)	124.2(6)
N(10)-C(38)-C(37)	116.2(6)
C(39)-C(38)-C(37)	119.6(6)
C(40)-C(39)-O(12)	120.7(6)
C(40)-C(39)-C(38)	118.8(7)
O(12)-C(39)-C(38)	120.4(6)
C(39)-C(40)-C(41)	120.1(7)
C(39)-C(40)-H(40)	120.0
C(41)-C(40)-H(40)	120.0
C(40)-C(41)-C(46)	123.7(8)
C(40)-C(41)-C(42)	117.7(6)
C(46)-C(41)-C(42)	118.6(8)
N(10)-C(42)-C(43)	120.0(7)
N(10)-C(42)-C(41)	121.3(7)
C(43)-C(42)-C(41)	118.7(7)
C(44)-C(43)-C(42)	122.1(10)
C(44)-C(43)-H(43)	118.9
C(42)-C(43)-H(43)	118.9
C(43)-C(44)-C(45)	118.5(10)
C(43)-C(44)-H(44)	120.8
C(45)-C(44)-H(44)	120.8
C(46)-C(45)-C(44)	123.3(8)
C(46)-C(45)-H(45)	118.4
C(44)-C(45)-H(45)	118.4
C(45)-C(46)-C(41)	118.9(9)
C(45)-C(46)-H(46)	120.5
C(41)-C(46)-H(46)	120.5
C(13')-S(1')-C(12')	99.4(4)
C(15')-S(2')-C(16')	97.5(3)
C(26')-S(3')-C(25')	100.6(4)
C(28')-S(4')-C(29')	101.2(5)
C(8')-O(1')-H(1')	109.5

C(39')-O(12')-H(12')	109.5	C(3')-C(2')-C(1')	121.5(9)
C(9')-N(1')-C(1')	117.5(7)	C(3')-C(2')-H(2'A)	119.2
C(10')-N(2')-C(11')	123.7(6)	C(1')-C(2')-H(2'A)	119.2
C(10')-N(2')-H(2')	118.2	C(2')-C(3')-C(4')	118.4(8)
C(11')-N(2')-H(2')	118.2	C(2')-C(3')-H(3'A)	120.8
C(36')-N(3')-C(35')	121.7(7)	C(4')-C(3')-H(3'A)	120.8
C(36')-N(3')-H(3')	119.2	C(5')-C(4')-C(3')	121.8(8)
C(35')-N(3')-H(3')	119.2	C(5')-C(4')-H(4')	119.1
C(34')-N(4')-C(30')	120.9(6)	C(3')-C(4')-H(4')	119.1
C(34')-N(4')-C(33')	121.7(7)	C(4')-C(5')-C(6')	121.1(9)
C(30')-N(4')-C(33')	117.3(6)	C(4')-C(5')-H(5')	119.5
C(31')-N(5')-C(27')	127.0(8)	C(6')-C(5')-H(5')	119.5
C(31')-N(5')-C(32')	117.4(9)	C(7')-C(6')-C(1')	119.6(8)
C(27')-N(5')-C(32')	115.6(8)	C(7')-C(6')-C(5')	123.3(8)
C(37')-N(6')-C(24')	122.5(7)	C(1')-C(6')-C(5')	117.1(8)
C(37')-N(6')-H(6')	118.7	C(6')-C(7')-C(8')	118.6(8)
C(24')-N(6')-H(6')	118.7	C(6')-C(7')-H(7'A)	120.7
C(23')-N(7')-C(22')	122.1(7)	C(8')-C(7')-H(7'A)	120.7
C(23')-N(7')-H(7')	118.9	O(1')-C(8')-C(7')	118.5(8)
C(22')-N(7')-H(7')	118.9	O(1')-C(8')-C(9')	121.7(8)
C(21')-N(8')-C(17')	119.0(6)	C(7')-C(8')-C(9')	119.8(8)
C(21')-N(8')-C(20')	122.8(7)	N(1')-C(9')-C(8')	123.1(7)
C(17')-N(8')-C(20')	118.1(6)	N(1')-C(9')-C(10')	116.7(7)
C(18')-N(9')-C(14')	126.8(6)	C(8')-C(9')-C(10')	120.1(7)
C(18')-N(9')-C(19')	117.5(6)	O(2')-C(10')-N(2')	122.9(7)
C(14')-N(9')-C(19')	115.7(6)	O(2')-C(10')-C(9')	119.0(7)
C(38')-N(10')-C(42')	119.2(8)	N(2')-C(10')-C(9')	118.1(6)
C(2')-C(1')-N(1')	118.5(8)	N(2')-C(11')-C(36')	106.6(6)
C(2')-C(1')-C(6')	120.1(8)	N(2')-C(11')-C(12')	110.1(6)
N(1')-C(1')-C(6')	121.3(7)	C(36')-C(11')-C(12')	108.9(6)

N(2')-C(11')-H(11')	110.4	N(8')-C(17')-C(18')	110.3(6)
C(36')-C(11')-H(11')	110.4	N(8')-C(17')-C(16')	110.6(6)
C(12')-C(11')-H(11')	110.4	C(18')-C(17')-C(16')	113.1(6)
C(11')-C(12')-S(1')	113.5(5)	N(8')-C(17')-H(17')	107.5
C(11')-C(12')-H(12A)	108.9	C(18')-C(17')-H(17')	107.5
S(1')-C(12')-H(12A)	108.9	C(16')-C(17')-H(17')	107.5
C(11')-C(12')-H(12B)	108.9	O(4')-C(18')-N(9')	122.5(7)
S(1')-C(12')-H(12B)	108.9	O(4')-C(18')-C(17')	120.1(7)
H(12A)-C(12')-H(12B)	107.7	N(9')-C(18')-C(17')	117.4(6)
O(3')-C(13')-C(14')	125.4(7)	N(9')-C(19')-H(19A)	109.5
O(3')-C(13')-S(1')	120.5(6)	N(9')-C(19')-H(19B)	109.5
C(14')-C(13')-S(1')	113.9(5)	H(19A)-C(19')-H(19B)	109.5
N(9')-C(14')-C(13')	112.9(6)	N(9')-C(19')-H(19C)	109.5
N(9')-C(14')-C(15')	118.1(6)	H(19A)-C(19')-H(19C)	109.5
C(13')-C(14')-C(15')	113.6(6)	H(19B)-C(19')-H(19C)	109.5
N(9')-C(14')-H(14')	103.3	N(8')-C(20')-H(20A)	109.5
C(13')-C(14')-H(14')	103.3	N(8')-C(20')-H(20B)	109.5
C(15')-C(14')-H(14')	103.3	H(20A)-C(20')-H(20B)	109.5
C(14')-C(15')-S(2')	117.7(5)	N(8')-C(20')-H(20C)	109.5
C(14')-C(15')-H(15A)	107.9	H(20A)-C(20')-H(20C)	109.5
S(2')-C(15')-H(15A)	107.9	H(20B)-C(20')-H(20C)	109.5
C(14')-C(15')-H(15B)	107.9	O(5')-C(21')-N(8')	122.2(7)
S(2')-C(15')-H(15B)	107.9	O(5')-C(21')-C(22')	120.4(7)
H(15A)-C(15')-H(15B)	107.2	N(8')-C(21')-C(22')	117.3(7)
C(17')-C(16')-S(2')	111.4(5)	N(7')-C(22')-C(21')	110.9(7)
C(17')-C(16')-H(16A)	109.3	N(7')-C(22')-H(22A)	109.5
S(2')-C(16')-H(16A)	109.3	C(21')-C(22')-H(22A)	109.5
C(17')-C(16')-H(16B)	109.3	N(7')-C(22')-H(22B)	109.5
S(2')-C(16')-H(16B)	109.3	C(21')-C(22')-H(22B)	109.5
H(16A)-C(16')-H(16B)	108.0	H(22A)-C(22')-H(22B)	108.1

O(6')-C(23')-N(7')	123.7(9)	C(30')-C(29')-S(4')	112.5(6)
O(6')-C(23')-C(24')	119.9(8)	C(30')-C(29')-H(29A)	109.1
N(7')-C(23')-C(24')	116.4(8)	S(4')-C(29')-H(29A)	109.1
N(6')-C(24')-C(23')	105.5(7)	C(30')-C(29')-H(29B)	109.1
N(6')-C(24')-C(25')	109.2(7)	S(4')-C(29')-H(29B)	109.1
C(23')-C(24')-C(25')	109.6(8)	H(29A)-C(29')-H(29B)	107.8
N(6')-C(24')-H(24')	110.8	N(4')-C(30')-C(29')	110.2(6)
C(23')-C(24')-H(24')	110.8	N(4')-C(30')-C(31')	109.3(7)
C(25')-C(24')-H(24')	110.8	C(29')-C(30')-C(31')	112.1(7)
C(24')-C(25')-S(3')	110.4(6)	N(4')-C(30')-H(30')	108.4
C(24')-C(25')-H(25A)	109.6	C(29')-C(30')-H(30')	108.4
S(3')-C(25')-H(25A)	109.6	C(31')-C(30')-H(30')	108.4
C(24')-C(25')-H(25B)	109.6	O(8')-C(31')-N(5')	123.1(8)
S(3')-C(25')-H(25B)	109.6	O(8')-C(31')-C(30')	119.2(8)
H(25A)-C(25')-H(25B)	108.1	N(5')-C(31')-C(30')	117.7(8)
O(7')-C(26')-C(27')	122.1(9)	N(5')-C(32')-H(32A)	109.5
O(7')-C(26')-S(3')	124.2(7)	N(5')-C(32')-H(32B)	109.5
C(27')-C(26')-S(3')	113.6(7)	H(32A)-C(32')-H(32B)	109.5
N(5')-C(27')-C(28')	115.3(8)	N(5')-C(32')-H(32C)	109.5
N(5')-C(27')-C(26')	112.7(7)	H(32A)-C(32')-H(32C)	109.5
C(28')-C(27')-C(26')	116.1(8)	H(32B)-C(32')-H(32C)	109.5
N(5')-C(27')-H(27')	103.5	N(4')-C(33')-H(33A)	109.5
C(28')-C(27')-H(27')	103.5	N(4')-C(33')-H(33B)	109.5
C(26')-C(27')-H(27')	103.5	H(33A)-C(33')-H(33B)	109.5
C(27')-C(28')-S(4')	116.5(6)	N(4')-C(33')-H(33C)	109.5
C(27')-C(28')-H(28A)	108.2	H(33A)-C(33')-H(33C)	109.5
S(4')-C(28')-H(28A)	108.2	H(33B)-C(33')-H(33C)	109.5
C(27')-C(28')-H(28B)	108.2	O(9')-C(34')-N(4')	122.5(7)
S(4')-C(28')-H(28B)	108.2	O(9')-C(34')-C(35')	121.4(7)
H(28A)-C(28')-H(28B)	107.3	N(4')-C(34')-C(35')	116.1(7)

N(3')-C(35')-C(34')	115.4(7)
N(3')-C(35')-H(35A)	108.4
C(34')-C(35')-H(35A)	108.4
N(3')-C(35')-H(35B)	108.4
C(34')-C(35')-H(35B)	108.4
H(35A)-C(35')-H(35B)	107.5
O(10')-C(36')-N(3')	122.7(8)
O(10')-C(36')-C(11')	119.2(7)
N(3')-C(36')-C(11')	117.9(6)
O(11')-C(37')-N(6')	123.3(8)
O(11')-C(37')-C(38')	120.8(8)
N(6')-C(37')-C(38')	115.9(7)
N(10')-C(38')-C(39')	122.0(8)
N(10')-C(38')-C(37')	117.2(7)
C(39')-C(38')-C(37')	120.8(7)
O(12')-C(39')-C(40')	121.8(10)
O(12')-C(39')-C(38')	119.9(9)
C(40')-C(39')-C(38')	118.3(9)
C(39')-C(40')-C(41')	120.3(9)
C(39')-C(40')-H(40')	119.8
C(41')-C(40')-H(40')	119.8
C(40')-C(41')-C(42')	118.3(9)
C(40')-C(41')-C(46')	122.4(10)
C(42')-C(41')-C(46')	119.2(10)
N(10')-C(42')-C(41')	121.8(8)
N(10')-C(42')-C(43')	118.6(8)
C(41')-C(42')-C(43')	119.6(9)
C(44')-C(43')-C(42')	120.3(11)
C(44')-C(43')-H(43')	119.9
C(42')-C(43')-H(43')	119.9
C(43')-C(44')-C(45')	119.3(11)
C(43')-C(44')-H(44')	120.3
C(45')-C(44')-H(44')	120.3
C(46')-C(45')-C(44')	123.1(11)
C(46')-C(45')-H(45')	118.5
C(44')-C(45')-H(45')	118.5
C(45')-C(46')-C(41')	118.6(12)
C(45')-C(46')-H(46')	120.7
C(41')-C(46')-H(46')	120.7
H(1SD)-O(1S)-H(1SE)	101(3)
C(1SM)-O(2S)-H(2S)	109.5
O(2S)-C(1SM)-H(1SA)	109.5
O(2S)-C(1SM)-H(1SB)	109.5
H(1SA)-C(1SM)-H(1SB)	109.5
O(2S)-C(1SM)-H(1SC)	109.5
H(1SA)-C(1SM)-H(1SC)	109.5
H(1SB)-C(1SM)-H(1SC)	109.5
C(2S)-C(1S)-H(1SF)	109.5
C(2S)-C(1S)-H(1SG)	109.5
H(1SF)-C(1S)-H(1SG)	109.5
C(2S)-C(1S)-H(1SH)	109.5
H(1SF)-C(1S)-H(1SH)	109.5
H(1SG)-C(1S)-H(1SH)	109.5
C(3S)-C(2S)-C(7S)	116.3(10)
C(3S)-C(2S)-C(1S)	123.4(12)
C(7S)-C(2S)-C(1S)	120.3(13)
C(4S)-C(3S)-C(2S)	124.2(11)
C(4S)-C(3S)-H(3S)	117.9
C(2S)-C(3S)-H(3S)	117.9
C(5S)-C(4S)-C(3S)	117.8(12)

C(5S)-C(4S)-H(4S)	121.1
C(3S)-C(4S)-H(4S)	121.1
C(4S)-C(5S)-C(6S)	122.3(11)
C(4S)-C(5S)-H(5S)	118.9
C(6S)-C(5S)-H(5S)	118.9
C(7S)-C(6S)-C(5S)	120.2(10)
C(7S)-C(6S)-H(6S)	119.9
C(5S)-C(6S)-H(6S)	119.9
C(6S)-C(7S)-C(2S)	119.2(11)
C(6S)-C(7S)-H(7S)	120.4
C(2S)-C(7S)-H(7S)	120.4
C(9S)-C(8S)-H(8SA)	109.5
C(9S)-C(8S)-H(8SB)	109.5
H(8SA)-C(8S)-H(8SB)	109.5
C(9S)-C(8S)-H(8SC)	109.5
H(8SA)-C(8S)-H(8SC)	109.5
H(8SB)-C(8S)-H(8SC)	109.5
C(10S)-C(9S)-C(8S)	125.6(18)
C(10S)-C(9S)-C(14S)	115.8(16)
C(8S)-C(9S)-C(14S)	118.4(16)
C(11S)-C(10S)-C(9S)	131(2)
C(11S)-C(10S)-H(10S)	114.6
C(9S)-C(10S)-H(10S)	114.6
C(10S)-C(11S)-C(12S)	110.0(18)
C(10S)-C(11S)-H(11S)	125.0
C(12S)-C(11S)-H(11S)	125.0
C(13S)-C(12S)-C(11S)	126.8(17)
C(13S)-C(12S)-H(12S)	116.6
C(11S)-C(12S)-H(12S)	116.6
C(12S)-C(13S)-C(14S)	118.4(18)
C(12S)-C(13S)-H(13S)	120.8
C(14S)-C(13S)-H(13S)	120.8
C(13S)-C(14S)-C(9S)	117.7(17)
C(13S)-C(14S)-H(14S)	121.1
C(9S)-C(14S)-H(14S)	121.1
C(16S)-C(15S)-H(15E)	109.5
C(16S)-C(15S)-H(15F)	109.5
H(15E)-C(15S)-H(15F)	109.5
C(16S)-C(15S)-H(15G)	109.5
H(15E)-C(15S)-H(15G)	109.5
H(15F)-C(15S)-H(15G)	109.5
C(17S)-C(16S)-C(21S)	122.4(18)
C(17S)-C(16S)-C(15S)	120(2)
C(21S)-C(16S)-C(15S)	118(2)
C(18S)-C(17S)-C(16S)	114.9(19)
C(18S)-C(17S)-H(17S)	122.6
C(16S)-C(17S)-H(17S)	122.6
C(17S)-C(18S)-C(19S)	119(2)
C(17S)-C(18S)-H(18S)	120.3
C(19S)-C(18S)-H(18S)	120.3
C(18S)-C(19S)-C(20S)	133(2)
C(18S)-C(19S)-H(19S)	113.5
C(20S)-C(19S)-H(19S)	113.5
C(19S)-C(20S)-C(21S)	108.4(19)
C(19S)-C(20S)-H(20S)	125.8
C(21S)-C(20S)-H(20S)	125.8
C(20S)-C(21S)-C(16S)	121.7(19)
C(20S)-C(21S)-H(21S)	119.2
C(16S)-C(21S)-H(21S)	119.2

Table S4. Anisotropic displacement parameters ($Å^2$x 10^3) for verrucosamide (**1**). The anisotropic displacement factor exponent takes the form: $-2\pi^2[h^2 a^{*2}U^{11} + ... + 2 h k a^* b^* U^{12}]$

	U^{11}	U^{22}	U^{33}	U^{23}	U^{13}	U^{12}
S(1)	44(1)	56(1)	63(1)	4(1)	-1(1)	2(1)
S(2)	70(1)	56(1)	70(1)	-5(1)	-23(1)	0(1)
S(3)	40(1)	51(1)	59(1)	-4(1)	1(1)	-2(1)
S(4)	47(1)	68(1)	62(1)	-2(1)	0(1)	7(1)
O(1)	69(4)	51(3)	60(3)	-10(3)	-14(3)	9(3)
O(2)	63(3)	51(3)	58(3)	-9(3)	-9(3)	9(3)
O(3)	55(3)	58(3)	101(5)	6(3)	-17(3)	0(3)
O(4)	54(3)	67(3)	81(4)	9(3)	-4(3)	14(3)
O(5)	48(3)	42(3)	63(3)	-11(2)	-7(2)	8(2)
O(6)	40(3)	61(3)	110(5)	-21(3)	10(3)	1(3)
O(7)	56(3)	74(4)	65(3)	6(3)	7(3)	24(3)
O(8)	48(3)	69(4)	107(5)	9(3)	-2(3)	15(3)
O(9)	42(3)	60(3)	44(3)	-11(2)	-13(2)	10(2)
O(10)	48(3)	77(4)	64(3)	12(3)	-19(3)	-13(3)
O(11)	51(3)	57(3)	49(3)	6(2)	-13(3)	0(2)
O(12)	73(4)	57(3)	45(3)	-4(2)	-1(3)	3(3)
N(1)	59(4)	57(4)	49(4)	3(3)	-5(3)	12(3)
N(2)	48(3)	54(4)	54(4)	-2(3)	6(3)	13(3)
N(3)	35(3)	60(4)	88(5)	8(4)	-4(3)	-7(3)
N(4)	48(3)	44(3)	62(4)	-8(3)	-1(3)	-2(3)
N(5)	40(3)	44(3)	66(4)	-9(3)	-6(3)	7(3)
N(6)	33(3)	48(3)	53(3)	-7(3)	-8(3)	2(3)
N(7)	44(3)	61(4)	69(4)	11(3)	3(3)	-2(3)
N(8)	37(3)	63(4)	66(4)	10(3)	-10(3)	10(3)
N(9)	33(3)	63(4)	50(3)	-8(3)	-4(3)	9(3)
N(10)	56(4)	39(3)	60(4)	-8(3)	-13(3)	-1(3)

C(1)	52(4)	66(5)	42(4)	10(4)	7(4)	1(4)
C(2)	74(6)	74(5)	50(5)	11(4)	3(4)	18(5)
C(3)	78(6)	73(6)	73(6)	28(5)	13(5)	15(5)
C(4)	53(5)	71(6)	105(8)	54(6)	-9(6)	0(5)
C(5)	57(5)	53(5)	82(6)	16(4)	-21(5)	-4(4)
C(6)	49(4)	55(4)	66(5)	22(4)	-11(4)	-9(4)
C(7)	67(5)	43(4)	60(5)	1(4)	-19(4)	-9(4)
C(8)	55(4)	45(4)	55(4)	8(3)	-7(4)	-17(4)
C(9)	49(4)	35(3)	46(4)	5(3)	-5(3)	-2(3)
C(10)	49(4)	44(4)	47(4)	0(3)	-1(3)	-8(3)
C(11)	54(4)	46(4)	58(5)	-5(3)	-11(4)	14(4)
C(12)	47(4)	64(5)	64(5)	-4(4)	3(4)	16(4)
C(13)	35(4)	63(5)	80(6)	17(4)	-7(4)	3(4)
C(14)	37(4)	57(4)	87(6)	9(4)	-14(4)	-8(3)
C(15)	47(4)	58(5)	99(7)	-5(5)	-17(5)	-9(4)
C(16)	58(5)	47(4)	63(5)	-5(4)	-6(4)	0(4)
C(17)	45(4)	43(4)	89(6)	-8(4)	-21(4)	-1(3)
C(18)	63(5)	38(4)	64(5)	-10(4)	-5(4)	7(4)
C(19)	64(6)	74(6)	107(8)	19(6)	19(6)	-11(5)
C(20)	58(5)	48(4)	76(6)	-11(4)	-4(4)	1(4)
C(21)	48(4)	45(4)	59(5)	1(3)	-11(4)	-3(4)
C(22)	54(4)	42(4)	56(4)	-8(3)	-6(4)	5(3)
C(23)	40(4)	46(4)	54(4)	-12(3)	5(3)	4(3)
C(24)	31(3)	55(4)	64(5)	-3(4)	-5(3)	2(3)
C(25)	40(4)	46(4)	53(4)	-14(3)	-3(3)	6(3)
C(26)	25(3)	51(4)	64(5)	-2(3)	-5(3)	-3(3)
C(27)	28(3)	53(4)	86(6)	8(4)	-5(4)	3(3)
C(28)	42(4)	80(6)	61(5)	22(4)	-1(4)	8(4)
C(29)	32(4)	77(5)	64(5)	7(4)	0(4)	4(4)
C(30)	37(4)	61(5)	53(4)	10(4)	-1(3)	-2(3)

C(31)	43(4)	66(5)	65(5)	9(4)	-10(4)	14(4)
C(32)	68(6)	60(5)	103(7)	15(5)	-9(6)	5(5)
C(33)	46(4)	83(6)	62(5)	23(4)	-4(4)	1(4)
C(34)	41(4)	55(4)	47(4)	-8(3)	3(4)	5(3)
C(35)	45(4)	60(4)	53(4)	-8(4)	-12(4)	1(4)
C(36)	42(4)	38(4)	56(4)	6(3)	-15(4)	0(3)
C(37)	35(4)	44(4)	54(4)	-3(3)	2(3)	-4(3)
C(38)	36(3)	32(3)	52(4)	3(3)	2(3)	-5(3)
C(39)	43(4)	43(4)	40(4)	-6(3)	-1(3)	-7(3)
C(40)	61(5)	42(4)	55(4)	-13(3)	11(4)	-6(4)
C(41)	33(3)	43(4)	73(5)	-6(4)	9(4)	-3(3)
C(42)	54(4)	40(4)	56(4)	-1(3)	-8(4)	-7(3)
C(43)	97(7)	49(5)	84(6)	-9(4)	-32(6)	-2(5)
C(44)	96(8)	53(5)	123(9)	10(6)	-56(7)	-2(5)
C(45)	49(5)	49(5)	140(10)	8(6)	-14(6)	2(4)
C(46)	53(5)	49(4)	90(6)	3(4)	9(5)	1(4)
S(1')	45(1)	44(1)	59(1)	-4(1)	-11(1)	2(1)
S(2')	56(1)	51(1)	56(1)	-1(1)	-11(1)	14(1)
S(3')	66(1)	63(1)	68(1)	-13(1)	-8(1)	13(1)
S(4')	59(1)	77(1)	74(1)	-26(1)	0(1)	5(1)
O(1')	60(4)	133(6)	86(5)	-60(5)	-16(4)	22(4)
O(2')	53(3)	89(4)	72(4)	-31(3)	-20(3)	14(3)
O(3')	36(3)	79(4)	117(5)	-41(4)	-24(3)	5(3)
O(4')	34(3)	55(3)	69(3)	5(2)	0(3)	8(2)
O(5')	72(4)	56(3)	54(3)	-8(2)	-14(3)	-13(3)
O(6')	150(7)	45(3)	103(5)	-28(3)	-48(5)	20(4)
O(7')	73(4)	87(4)	57(3)	-11(3)	-10(3)	16(3)
O(8')	45(3)	71(4)	98(5)	3(3)	-24(3)	-1(3)
O(9')	48(3)	49(3)	59(3)	-10(2)	-5(3)	12(2)
O(10')	50(3)	63(4)	108(5)	-37(3)	-10(3)	-4(3)

O(11')	90(4)	45(3)	67(4)	-7(3)	0(3)	-1(3)
O(12')	97(5)	68(4)	117(6)	-12(4)	-3(5)	-11(4)
N(1')	55(4)	51(3)	57(4)	1(3)	-2(3)	-7(3)
N(2')	50(4)	46(3)	54(3)	-12(3)	-18(3)	2(3)
N(3')	48(4)	51(3)	65(4)	-6(3)	-3(3)	-5(3)
N(4')	41(3)	47(3)	72(4)	-11(3)	-6(3)	7(3)
N(5')	54(4)	62(4)	85(5)	-8(4)	14(4)	6(3)
N(6')	79(5)	48(4)	56(4)	-5(3)	4(4)	15(4)
N(7')	81(5)	51(4)	49(4)	-6(3)	-3(4)	13(4)
N(8')	51(4)	43(3)	51(4)	8(3)	-12(3)	-8(3)
N(9')	46(3)	42(3)	47(3)	-1(3)	-12(3)	2(3)
N(10')	71(5)	46(4)	65(4)	5(3)	8(4)	6(3)
C(1')	46(4)	68(5)	47(4)	4(4)	-7(4)	-12(4)
C(2')	61(5)	45(4)	67(5)	0(4)	-8(4)	-7(4)
C(3')	67(5)	51(4)	58(5)	0(4)	-3(4)	-13(4)
C(4')	59(5)	90(6)	50(5)	1(5)	-7(4)	-17(5)
C(5')	35(4)	116(8)	66(5)	-4(6)	0(4)	-20(5)
C(6')	48(5)	79(6)	56(5)	9(4)	-6(4)	-3(4)
C(7')	41(5)	107(7)	77(6)	-11(6)	-5(5)	18(5)
C(8')	45(5)	93(7)	76(6)	-27(5)	-2(4)	2(4)
C(9')	43(4)	73(5)	48(4)	-9(4)	-8(4)	-4(4)
C(10')	46(4)	55(4)	50(4)	-13(4)	-15(4)	8(4)
C(11')	45(4)	36(3)	61(5)	-6(3)	-9(4)	-8(3)
C(12')	46(4)	44(4)	67(5)	-1(4)	-8(4)	-3(3)
C(13')	36(4)	60(4)	57(5)	0(4)	1(4)	-6(3)
C(14')	39(4)	57(4)	34(3)	4(3)	-5(3)	-3(3)
C(15')	42(4)	58(4)	39(4)	-3(3)	-9(3)	0(3)
C(16')	57(4)	38(4)	55(4)	7(3)	-18(4)	-6(3)
C(17')	40(4)	38(3)	53(4)	4(3)	-11(3)	1(3)
C(18')	50(4)	41(4)	41(4)	-5(3)	-7(3)	-6(3)

C(19')	57(5)	58(4)	57(5)	21(4)	-7(4)	4(4)
C(20')	55(5)	61(5)	51(4)	13(4)	-8(4)	-18(4)
C(21')	58(5)	44(4)	50(4)	-2(3)	-10(4)	-3(4)
C(22')	76(6)	64(5)	50(4)	-1(4)	-3(4)	-3(4)
C(23')	78(6)	79(6)	46(5)	-8(4)	5(4)	16(5)
C(24')	81(6)	48(4)	64(5)	-2(4)	2(5)	12(4)
C(25')	73(6)	68(5)	60(5)	-8(4)	-5(4)	23(5)
C(26')	58(5)	69(5)	57(5)	10(4)	8(4)	7(4)
C(27')	52(5)	75(6)	70(6)	8(4)	7(4)	14(4)
C(28')	63(5)	72(6)	83(6)	-15(5)	20(5)	13(4)
C(29')	44(4)	56(4)	83(6)	-21(4)	4(4)	4(4)
C(30')	36(4)	56(4)	64(5)	-12(4)	-2(4)	6(3)
C(31')	47(4)	51(4)	85(6)	-4(4)	1(5)	8(4)
C(32')	56(5)	91(7)	119(9)	-1(6)	10(6)	-6(5)
C(33')	47(4)	59(5)	86(6)	-7(4)	-9(4)	10(4)
C(34')	41(4)	46(4)	57(4)	-8(3)	-9(4)	-7(4)
C(35')	41(4)	54(5)	89(6)	-4(4)	-4(4)	2(3)
C(36')	50(4)	40(4)	57(4)	-3(3)	-21(4)	3(3)
C(37')	65(5)	45(4)	61(5)	-6(4)	8(4)	2(4)
C(38')	69(5)	41(4)	58(5)	6(4)	-4(4)	-6(4)
C(39')	84(7)	48(5)	85(7)	5(4)	17(5)	-9(5)
C(40')	61(6)	79(6)	95(7)	10(6)	-4(5)	-9(5)
C(41')	69(6)	64(5)	80(6)	10(5)	1(5)	10(5)
C(42')	62(5)	52(5)	79(6)	15(4)	8(5)	5(4)
C(43')	91(7)	50(5)	104(8)	1(5)	14(6)	7(5)
C(44')	121(10)	50(5)	121(9)	0(5)	17(8)	34(6)
C(45')	80(7)	78(7)	136(11)	2(7)	1(7)	14(6)
C(46')	94(8)	104(9)	90(8)	10(7)	-5(7)	8(7)
O(1S)	92(5)	99(5)	71(4)	5(4)	7(4)	10(4)
O(2S)	180(11)	184(10)	302(19)	-94(13)	-37(11)	-26(9)

C(1SM)	180(11)	184(10)	302(19)	-94(13)	-37(11)	-26(9)
C(1S)	199(16)	85(8)	95(9)	8(7)	-7(10)	39(10)
C(2S)	143(11)	62(5)	57(5)	-8(4)	-7(7)	10(7)
C(3S)	129(11)	85(7)	57(6)	-3(5)	21(6)	-15(7)
C(4S)	102(8)	89(8)	79(7)	-13(6)	11(6)	4(7)
C(5S)	107(9)	75(7)	80(7)	-7(5)	-17(6)	22(6)
C(6S)	116(9)	66(6)	64(6)	-6(5)	-5(6)	-1(6)
C(7S)	95(7)	83(7)	63(6)	-20(5)	-11(5)	-6(6)
C(8S)	138(5)	151(6)	146(5)	-26(4)	2(5)	-31(4)
C(9S)	138(5)	151(6)	146(5)	-26(4)	2(5)	-31(4)
C(10S)	138(5)	151(6)	146(5)	-26(4)	2(5)	-31(4)
C(11S)	138(5)	151(6)	146(5)	-26(4)	2(5)	-31(4)
C(12S)	138(5)	151(6)	146(5)	-26(4)	2(5)	-31(4)
C(13S)	138(5)	151(6)	146(5)	-26(4)	2(5)	-31(4)
C(14S)	138(5)	151(6)	146(5)	-26(4)	2(5)	-31(4)
C(15S)	203(5)	195(5)	201(5)	10(4)	11(4)	-1(4)
C(16S)	203(5)	195(5)	201(5)	10(4)	11(4)	-1(4)
C(17S)	203(5)	195(5)	201(5)	10(4)	11(4)	-1(4)
C(18S)	203(5)	195(5)	201(5)	10(4)	11(4)	-1(4)
C(19S)	203(5)	195(5)	201(5)	10(4)	11(4)	-1(4)
C(20S)	203(5)	195(5)	201(5)	10(4)	11(4)	-1(4)
C(21S)	203(5)	195(5)	201(5)	10(4)	11(4)	-1(4)

Table S5. Hydrogen coordinates (x 10^4) and isotropic displacement parameters ($Å^2$x 10^3) for verrucosamide (**1**).

	x	y	z	U(eq)
H(1)	8149	16978	10676	90
H(12)	10498	17353	10520	88
H(2)	8161	15712	9761	62
H(5)	9913	15415	9461	60
H(6)	10200	16453	9539	54
H(9)	8464	16554	9336	58
H(2A)	9776	14291	9988	79
H(3)	11005	13793	10194	89
H(4)	11669	14356	10654	92
H(5A)	11060	15282	10942	77
H(7)	9846	16165	10996	68
H(11)	6959	16764	9702	63
H(12C)	5915	15923	9546	70
H(12D)	6182	15873	9936	70
H(14)	5417	13630	9288	72
H(15C)	5363	13938	8745	81
H(15D)	5861	13194	8770	81
H(16C)	7407	13074	8726	67
H(16D)	8012	13663	8562	67
H(17)	7623	14356	9100	71
H(19D)	6672	12538	9714	122
H(19E)	5762	12631	9562	122
H(19F)	6095	13188	9842	122
H(20D)	9408	13329	8762	91
H(20E)	8824	12782	8967	91

H(20F)	9633	13108	9144	91
H(22C)	10165	13991	9459	61
H(22D)	10250	14273	9076	61
H(24)	11432	15775	9897	60
H(25C)	12431	15940	9438	55
H(25D)	12303	16589	9703	55
H(27)	12815	17246	8523	67
H(28C)	12956	16289	8198	73
H(28D)	12371	16841	7998	73
H(29C)	10774	16722	7945	69
H(29D)	10254	16006	8033	69
H(30)	10701	16310	8631	61
H(32D)	11481	18603	8527	115
H(32E)	12398	18370	8423	115
H(32F)	12107	18310	8809	115
H(33D)	9461	17229	8048	95
H(33E)	8619	17203	8259	95
H(33F)	8923	16499	8056	95
H(35C)	8136	17095	8750	63
H(35D)	8068	16236	8683	63
H(40)	9234	18774	10430	63
H(43)	8712	17996	9153	92
H(44)	7896	18998	9089	109
H(45)	7686	19779	9553	95
H(46)	8284	19579	10070	77
H(1')	8614	12963	7105	140
H(12')	7420	11970	8983	141
H(2')	6832	14132	7690	60
H(3')	4587	13144	7632	66
H(6')	5631	10616	8501	73

H(7')	3793	12016	8229	72
H(2'A)	8390	15003	8359	69
H(3'A)	9527	15215	8696	70
H(4')	10793	14656	8556	80
H(5')	10915	13938	8096	87
H(7'A)	10250	13339	7590	90
H(11')	5909	13031	7425	57
H(12A)	6308	13750	6918	63
H(12B)	5759	14397	7069	63
H(14')	4871	12685	6164	52
H(15A)	5648	11700	6124	56
H(15B)	4719	11428	6084	56
H(16A)	3873	10825	6536	60
H(16B)	4210	10450	6876	60
H(17')	4562	11805	7003	53
H(19A)	3468	12737	6000	85
H(19B)	3692	13418	6235	85
H(19C)	2885	12945	6313	85
H(20A)	3046	10397	7301	84
H(20B)	2753	10803	6963	84
H(20C)	2408	11060	7323	84
H(22A)	2830	11563	7856	76
H(22B)	3355	10826	7834	76
H(24')	4911	11960	8576	77
H(25A)	4743	11387	9141	80
H(25B)	4264	10725	8964	80
H(27')	2563	12503	9595	79
H(28A)	3282	13303	9871	87
H(28B)	2581	13756	9681	87
H(29A)	2848	14543	9121	73

H(29B)	3743	14715	8969	73
H(30')	3757	13323	8884	62
H(32A)	1100	12872	9037	133
H(32B)	1278	12769	9433	133
H(32C)	1480	12124	9173	133
H(33A)	3275	14941	8286	96
H(33B)	2579	14728	8556	96
H(33C)	2551	14393	8182	96
H(35A)	3490	13838	7809	73
H(35B)	4132	14423	7949	73
H(40')	9028	10883	8838	95
H(43')	7086	9123	8133	98
H(44')	8238	8445	8004	117
H(45')	9541	8816	8205	118
H(46')	9710	9827	8534	115
H(1SD)	9300(80)	13070(50)	9950(20)	131
H(1SE)	9440(90)	12370(20)	9830(30)	131
H(2S)	7430	17413	6933	333
H(1SA)	8287	17354	7443	333
H(1SB)	7499	17769	7593	333
H(1SC)	8281	18223	7465	333
H(1SF)	14092	15905	7643	190
H(1SG)	14310	15538	7286	190
H(1SH)	13547	15243	7504	190
H(3S)	12222	15706	7365	109
H(4S)	11362	16515	7099	108
H(5S)	11924	17518	6840	105
H(6S)	13328	17743	6852	98
H(7S)	14220	16924	7114	97
H(8SA)	4321	14163	10480	217

H(8SB)	4985	13521	10505	217
H(8SC)	4865	13952	10156	217
H(10S)	4777	15330	10679	174
H(11S)	5704	16195	10838	174
H(12S)	7136	15765	10833	174
H(13S)	7561	14712	10564	174
H(14S)	6545	13839	10401	174
H(15E)	9267	17494	6770	300
H(15F)	9706	17080	6463	300
H(15G)	10150	17123	6824	300
H(17S)	9815	16274	7276	240
H(18S)	9161	15172	7386	240
H(19S)	8297	14708	7016	240
H(20S)	7830	15197	6509	240
H(21S)	8548	16310	6362	240

Table S6. Hydrogen bonds for verrucosamide (**1**) [Å and °].

D-H...A	d(D-H)	d(H...A)	d(D...A)	<(DHA)
O(1)-H(1)...O(2)	0.84	1.91	2.629(8)	143.0
O(12)-H(12)...O(11)	0.84	1.79	2.526(8)	145.6
N(6)-H(6)...O(9)	0.88	2.13	2.960(7)	158.1
O(1')-H(1')...O(2')	0.84	1.85	2.545(8)	138.9
O(12')-H(12')...O(11')	0.84	1.86	2.582(11)	143.6
N(3')-H(3')...O(5')	0.88	2.01	2.869(8)	165.5
N(7')-H(7')...O(9')	0.88	2.14	2.938(8)	149.7

Table S7. NMR spectroscopic data for compounds **5** and **6**.

No.	**5**		**6**	
	δ_C, mult[a]	δ_H, mult (*J* in Hz)[b]	δ_C, mult[a]	δ_H, mult (*J* in Hz)[b]
2	145.5	-	145.9	-
3	151.0	-	151.3	-
4	115.7	8.02, br s	120.9	7.88, s
4a	131.1		Not observed-	-
5	127.9	8.09, br d (8.7)	126.9	7.69, m[c]
6	129.2	7.72, m[c]	128.2	8.06, m
7	129.9	7.73, m[c]	129.9	7.92, m
8	127.0	8.00, br d (7.3)	132.4	7.68, m[c]
8a	141.7	-	142.2	-
9	191.5	-	Not visible	-
10	55.7	4.06, s	-	10.3, s (OH)
11	11.2	2.47, s	10.9	2.49, s

Figure S1. ^{1}H NMR spectrum (500 MHz, acetone-d_6) of verrucosamide (**1**)

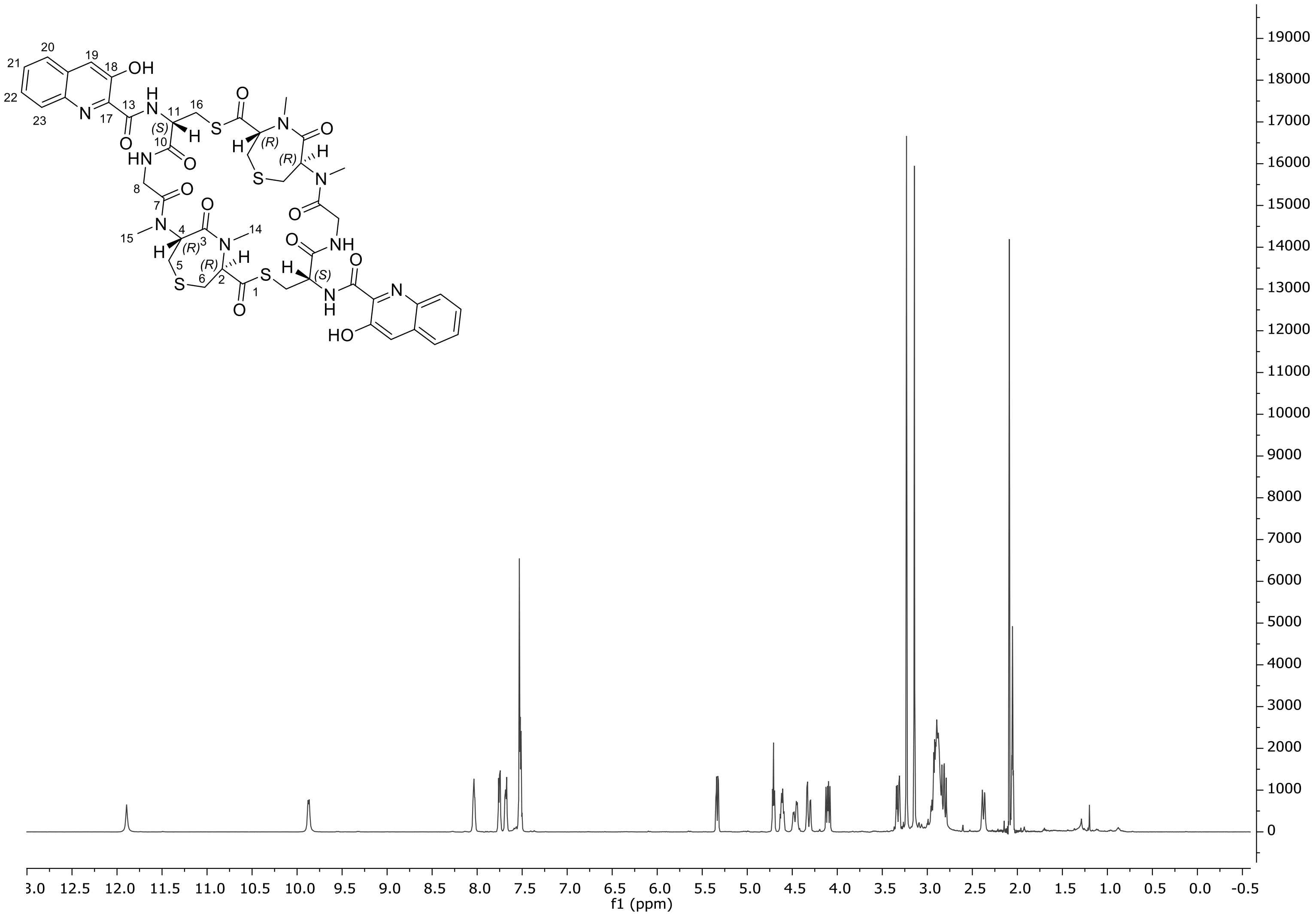

Figure S2. ^{13}C NMR spectrum (125 MHz, acetone-d_6) of verrucosamide (**1**)

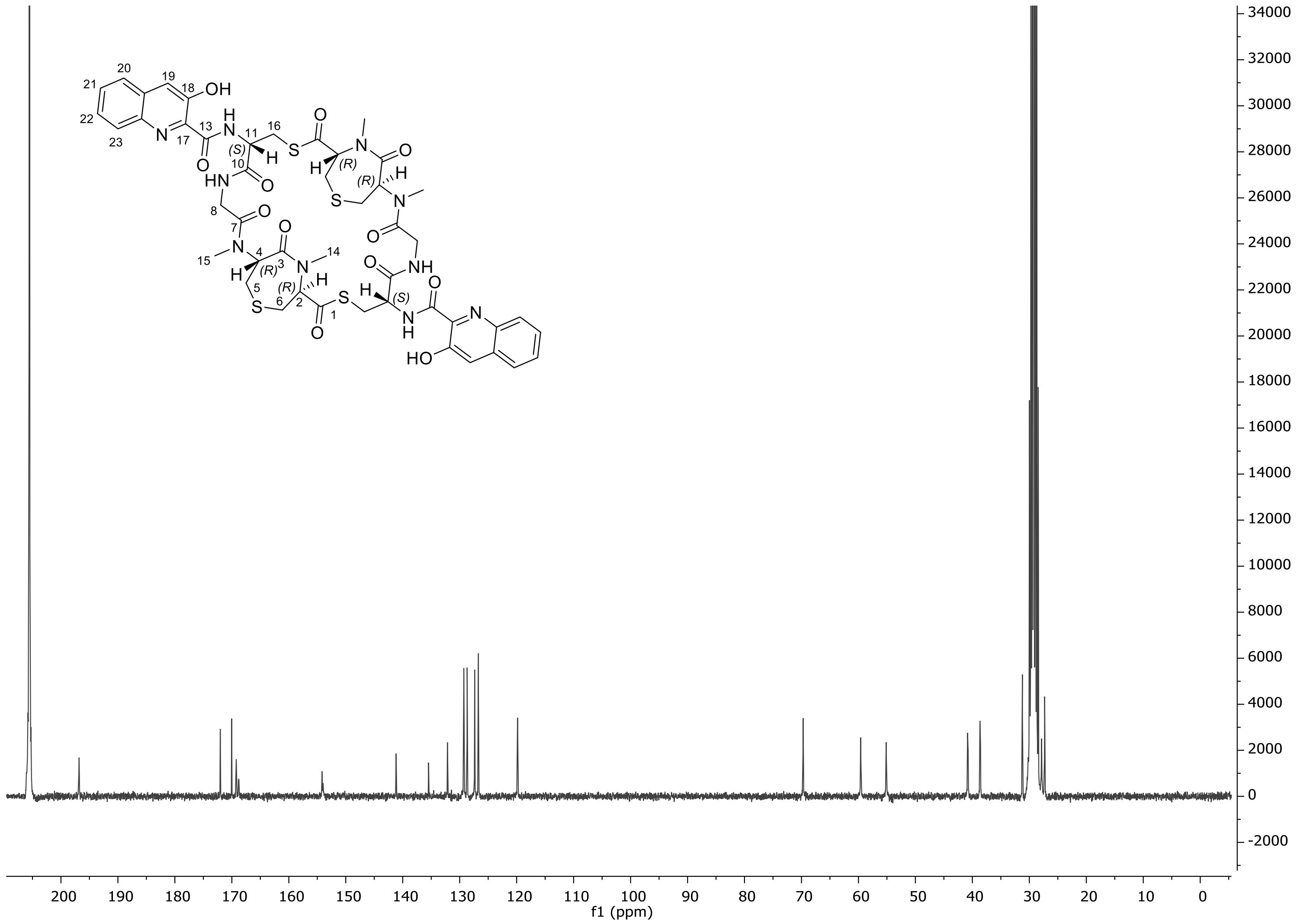

Figure S3. COSY NMR spectrum (500 MHz, acetone-d_6) of verrucosamide (**1**)

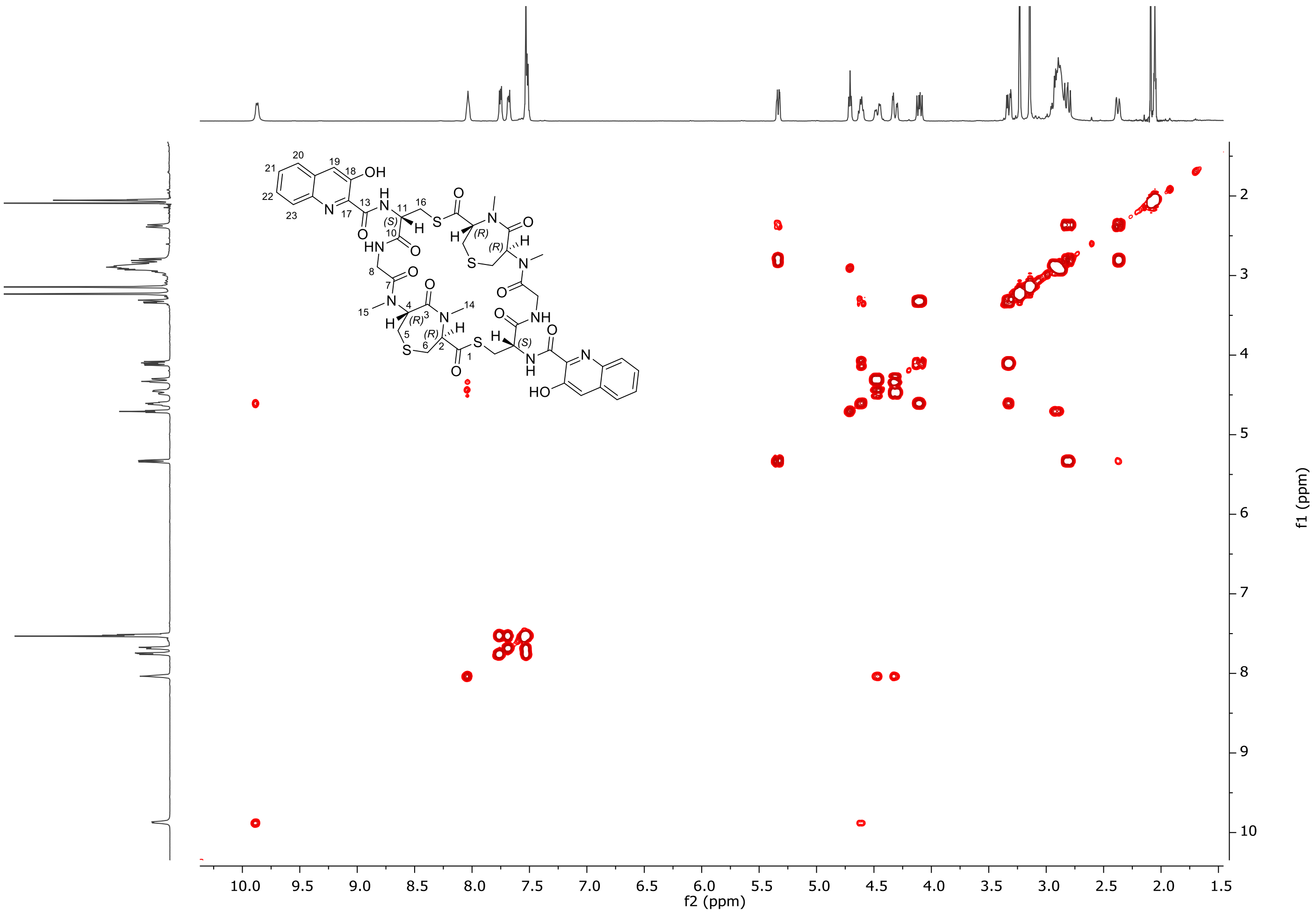

Figure S4. HSQC NMR spectrum (500 MHz, acetone-d_6) of verrucosamide (**1**)

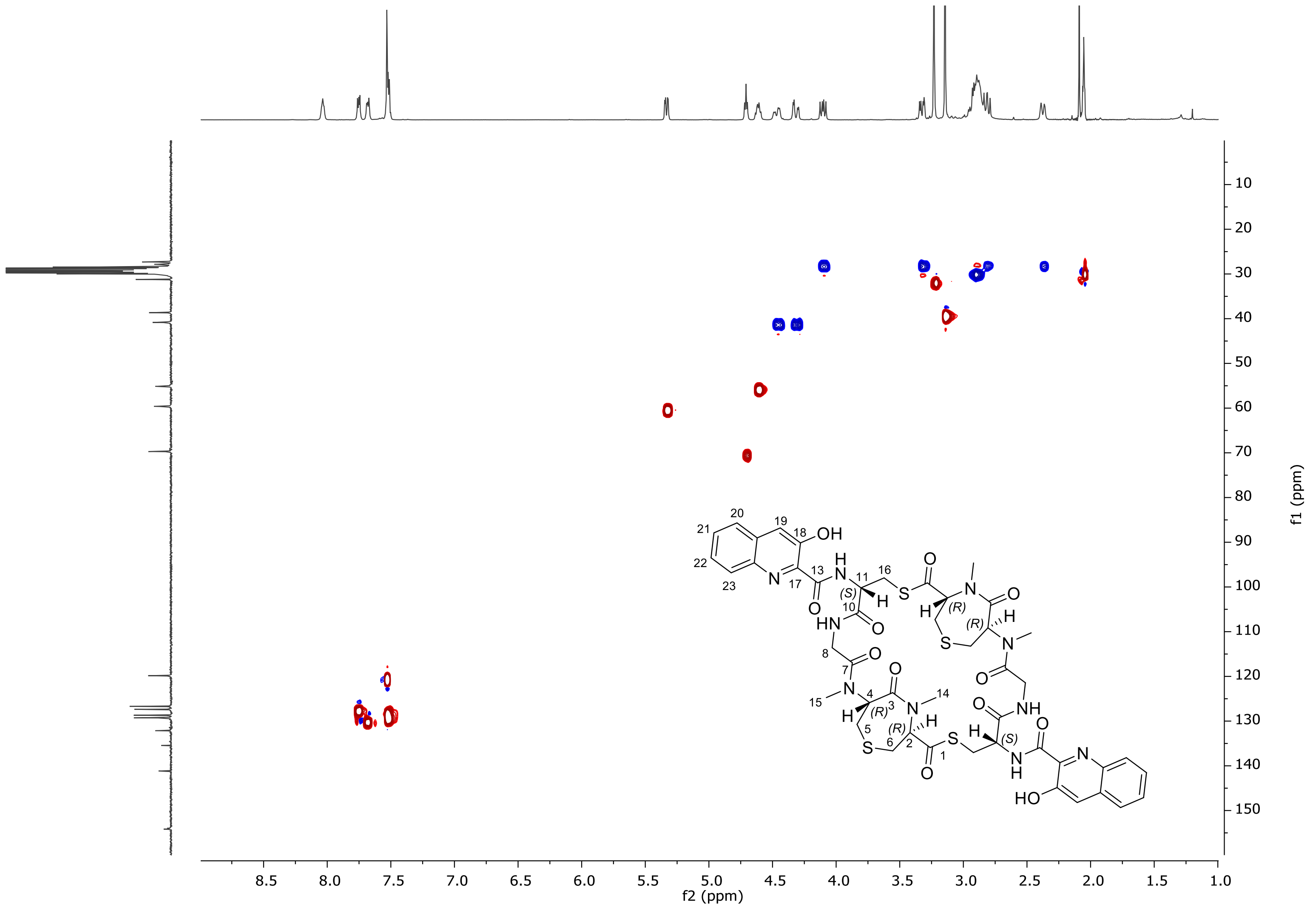

Figure S5. HMBC NMR spectrum (500 MHz, acetone-d_6) of verrucosamide (**1**)

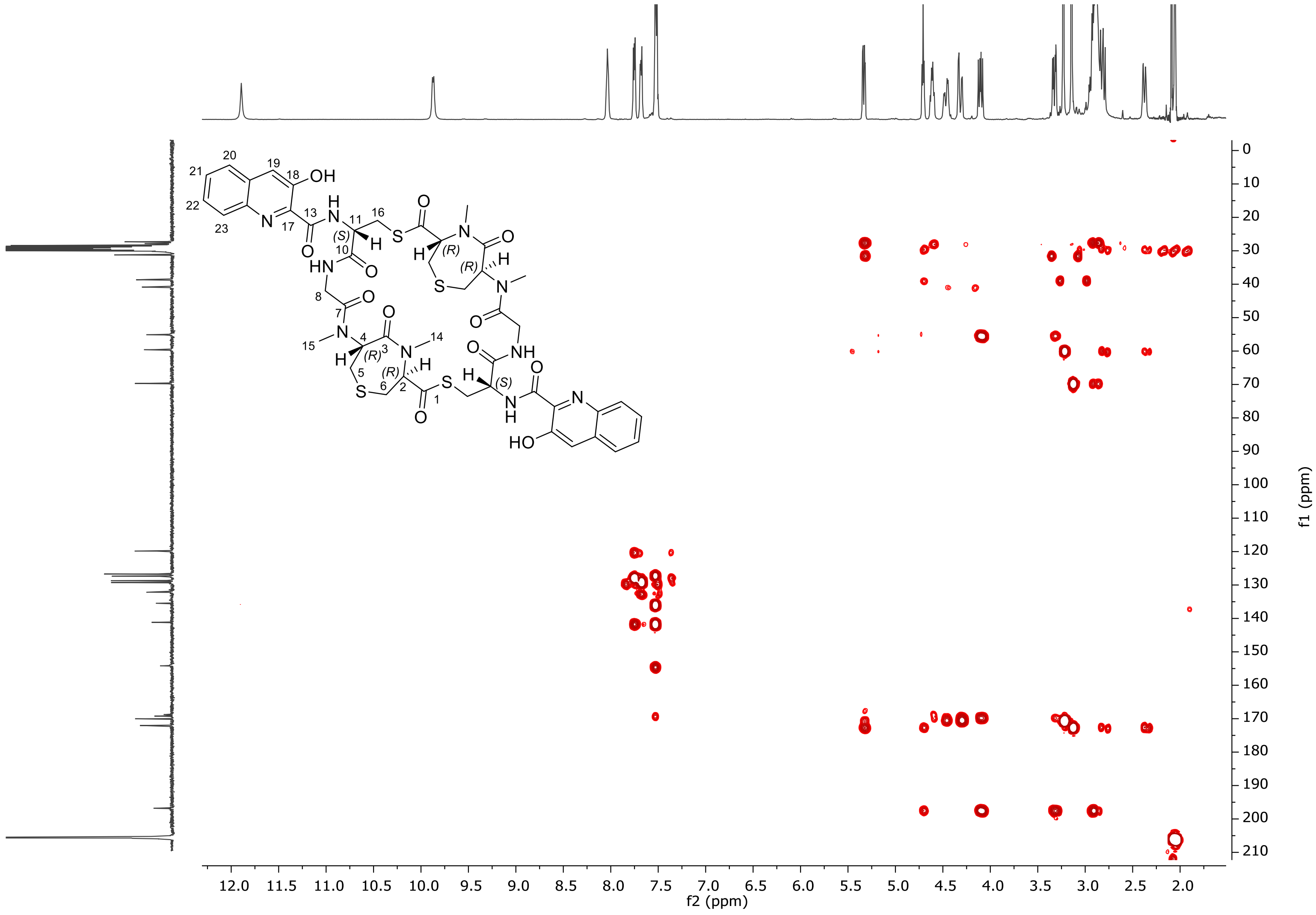

Figure S6. ROESY NMR spectrum (500 MHz, acetone-d_6) of verrucosamide (**1**)

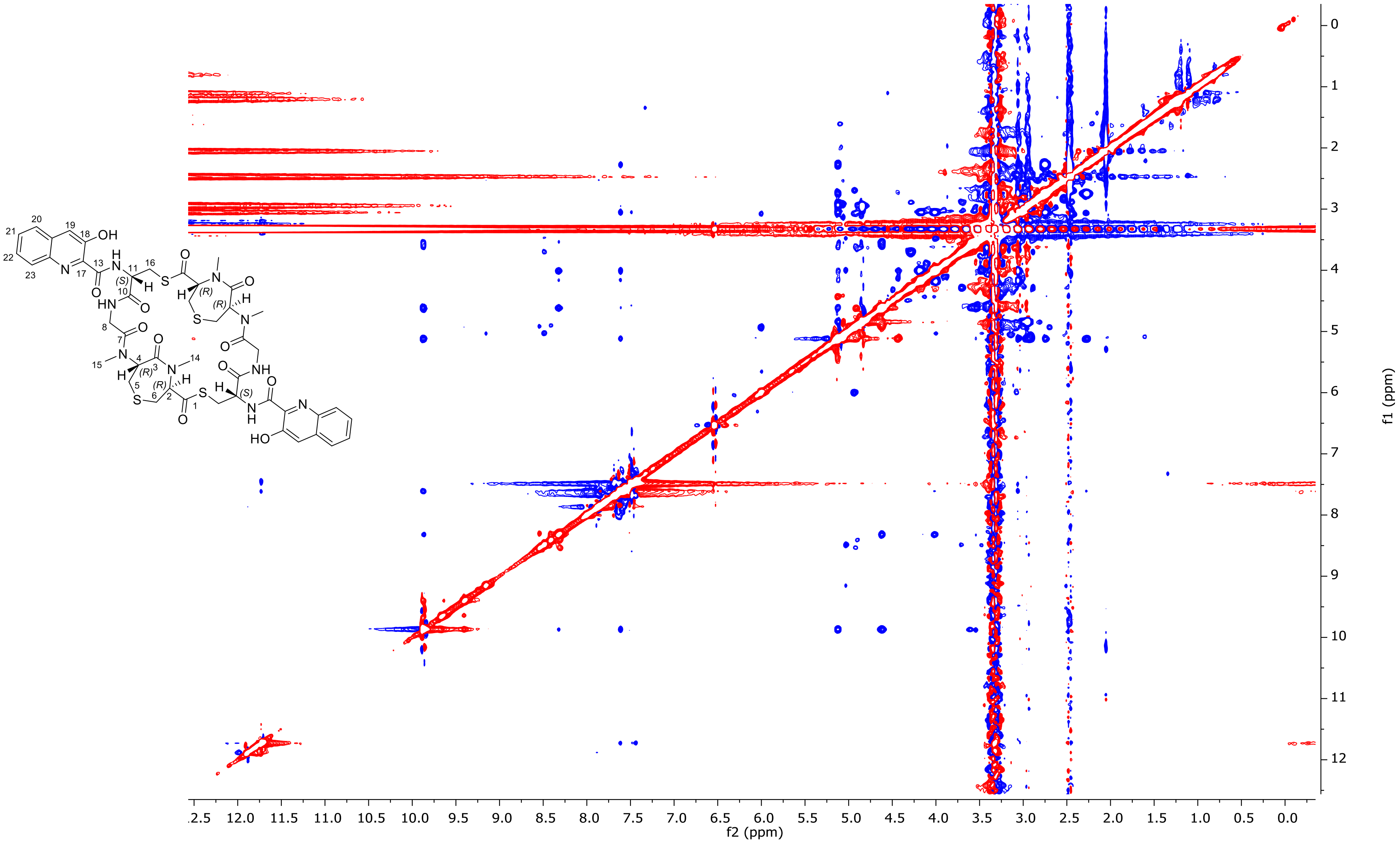

Figure S7. ^{1}H NMR spectrum (500 MHz, acetone-d_6) of compound **5**

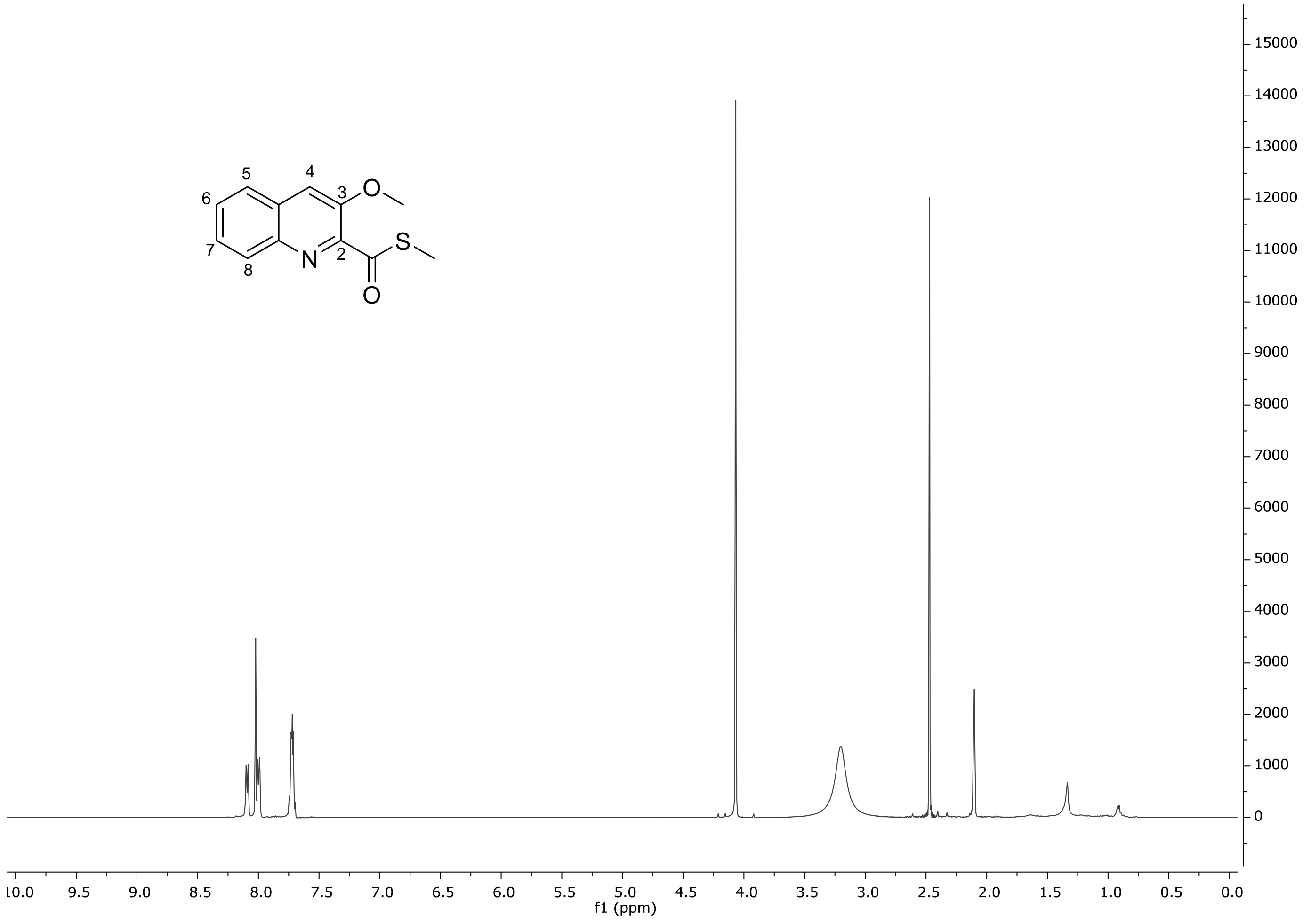

Figure S8. ^{13}C NMR spectrum (125 MHz, acetone-d_6) of compound **5**.

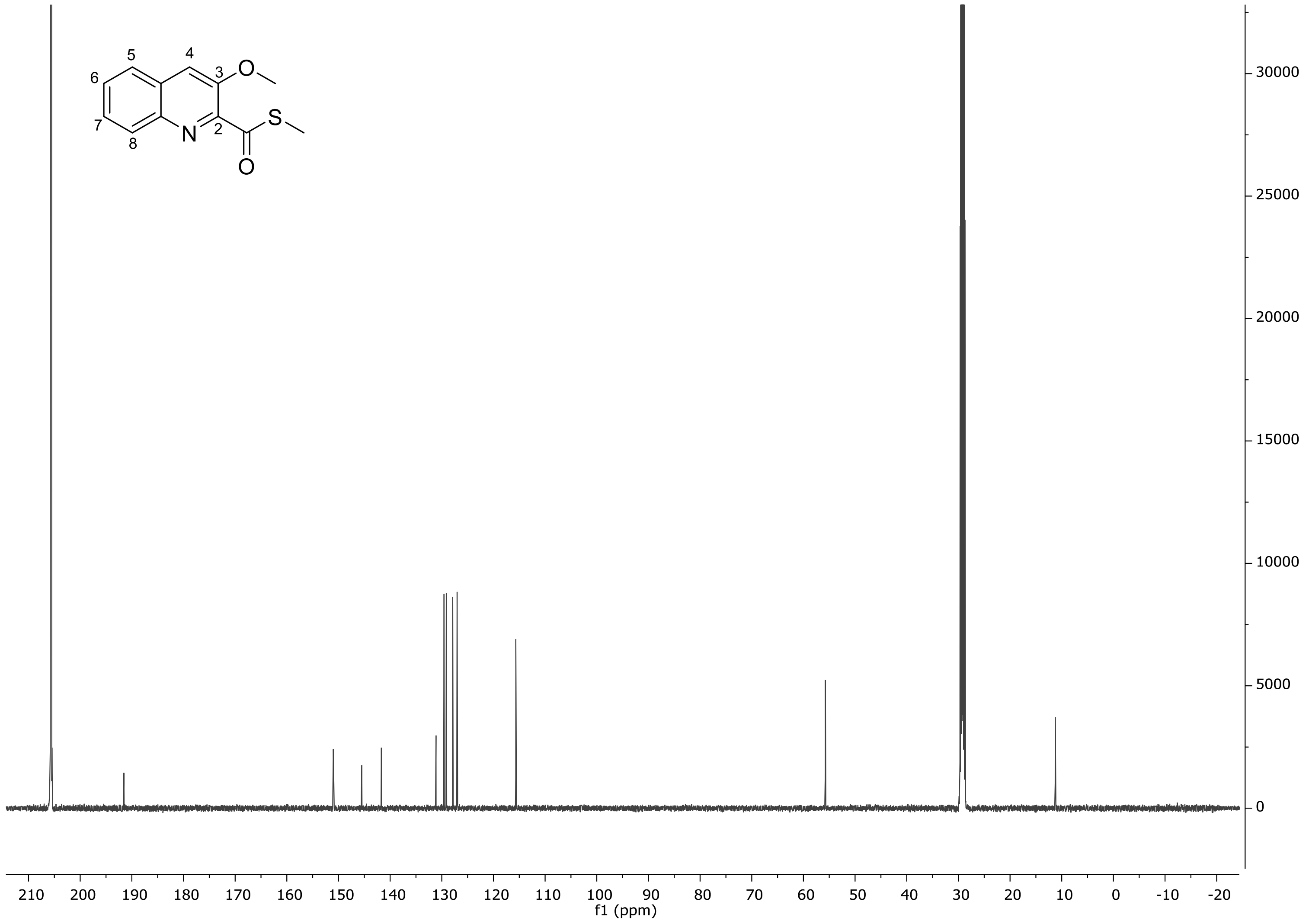

Figure S9. ^{1}H NMR spectrum (500 MHz, acetone-d_6) of compound **6**.

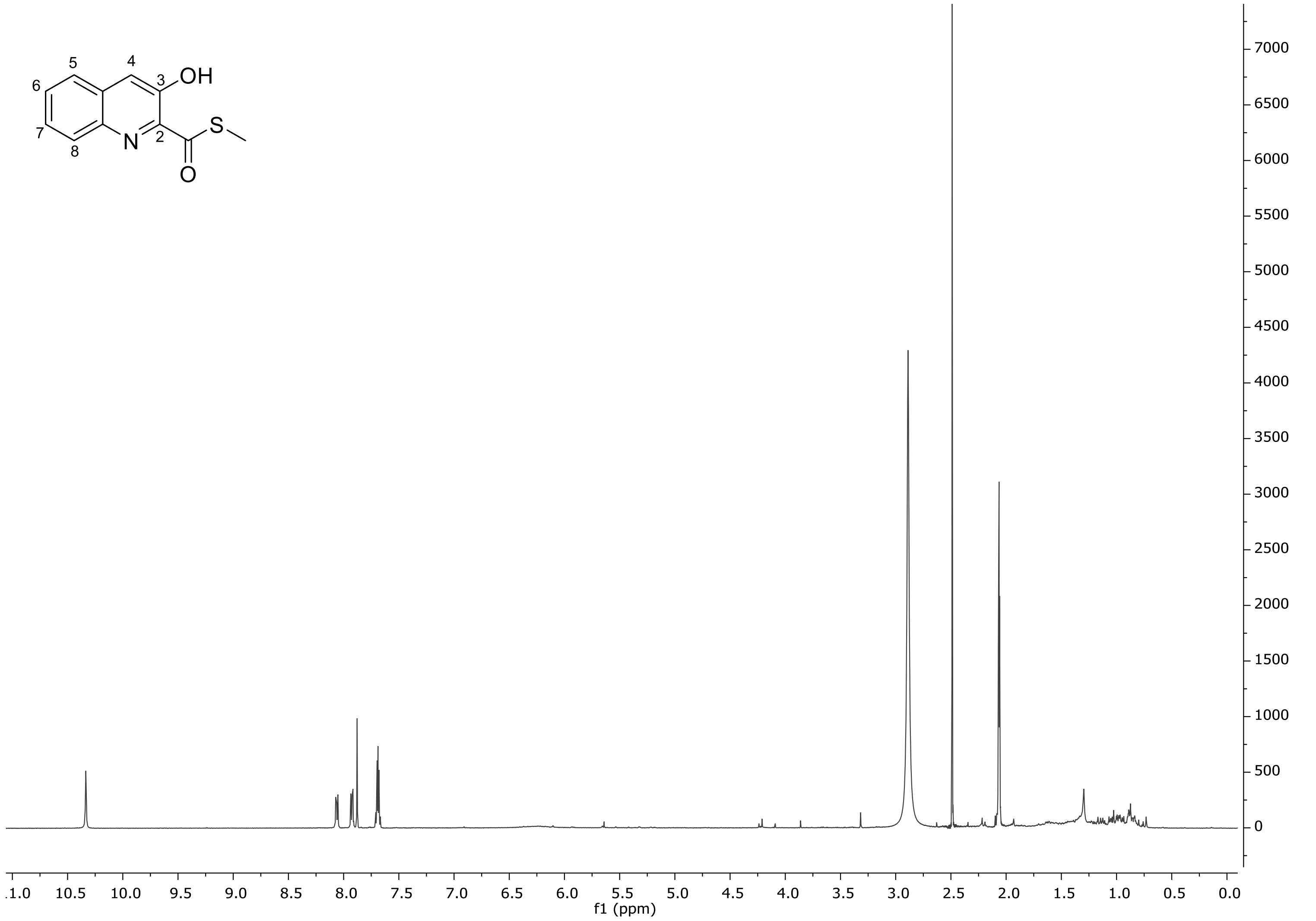

Figure S10. ^{13}C NMR spectrum (125 MHz, acetone-d_6) of compound **6**.

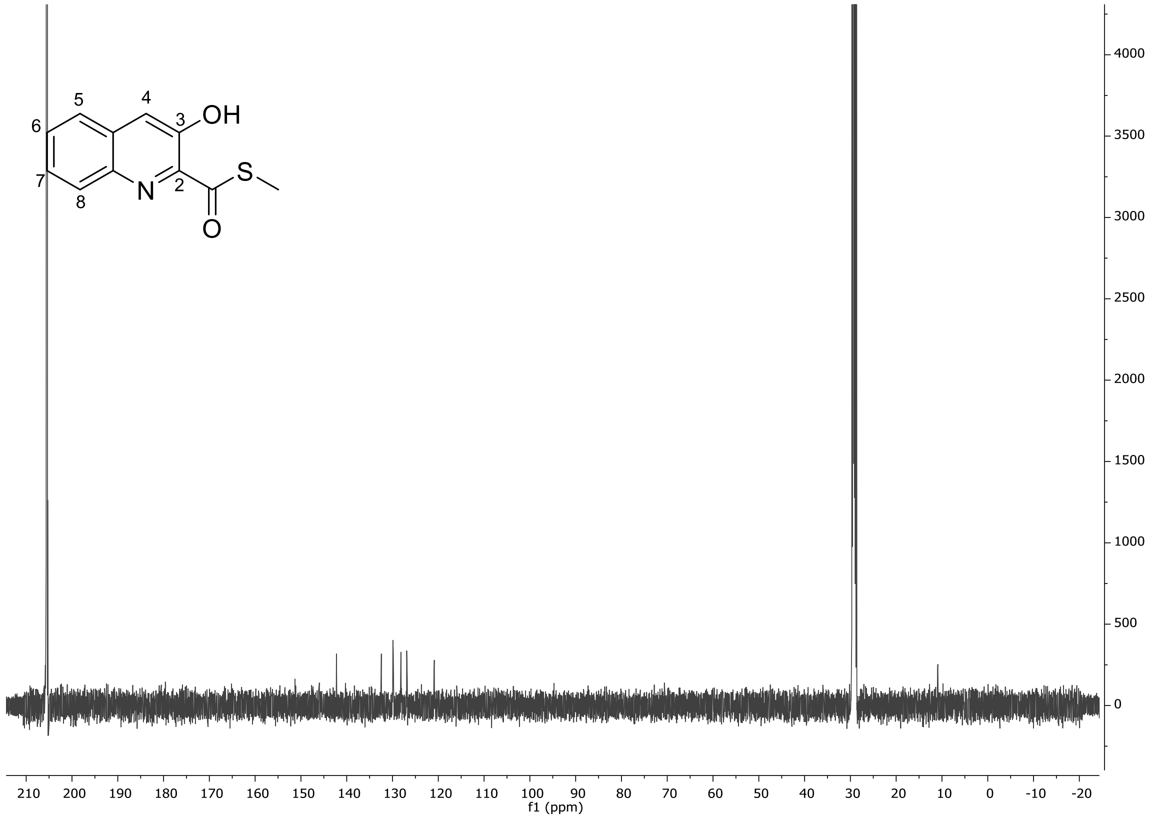

National Cancer Institute Developmental Therapeutics Program Dose Response Curves	NSC : D - 763852/1	SSPL:075T	EXP. ID:1203NS37
	Report Date:July 29, 2012	Test Date:March 26, 2012	

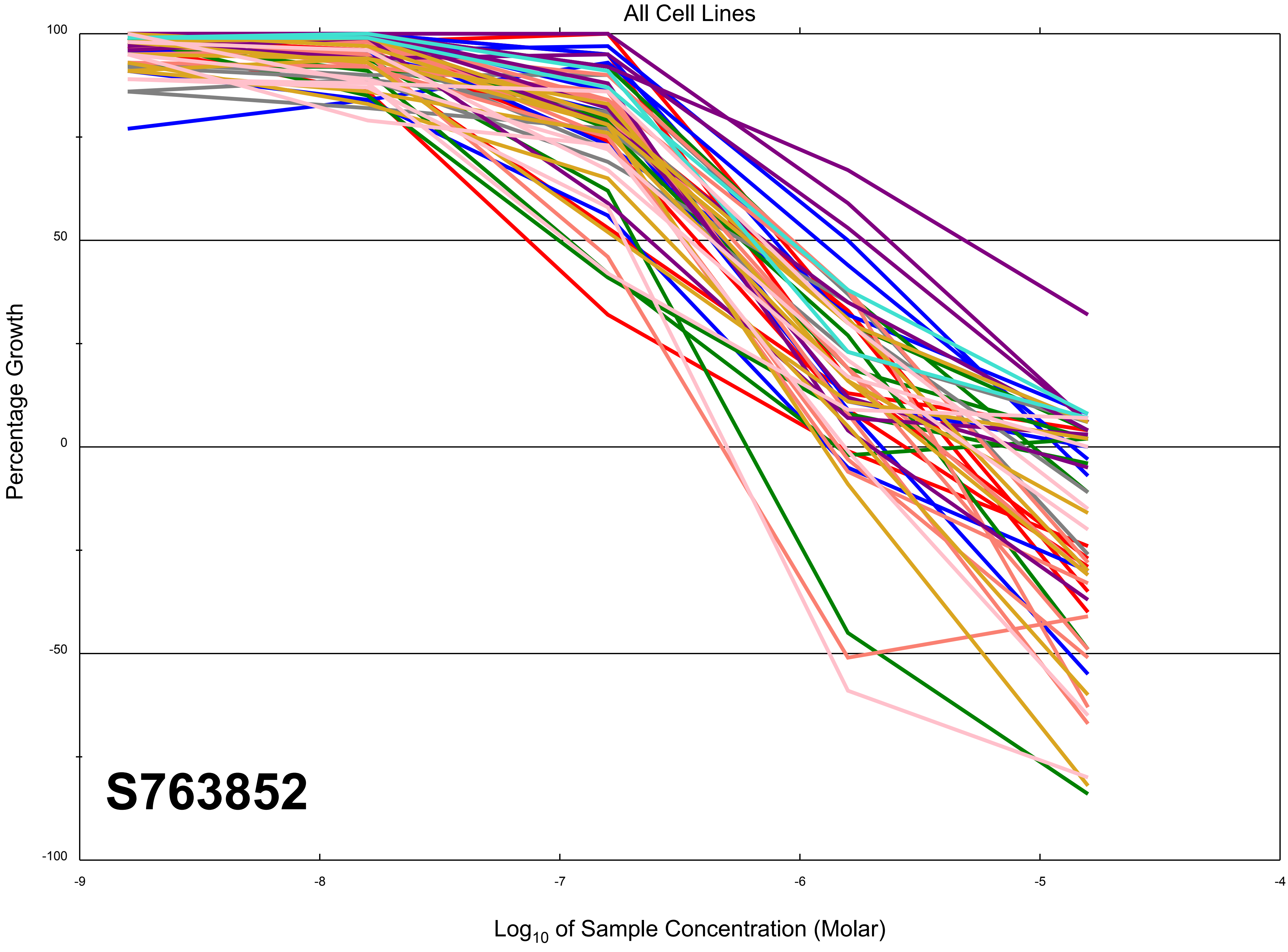

National Cancer Institute Developmental Therapeutics Program
Dose Response Curves

NSC: D - 763852 / 1 | SSPL: 075T | EXP. ID: 1203NS37

Report Date: July 29, 2012 | Test Date: March 26, 2012

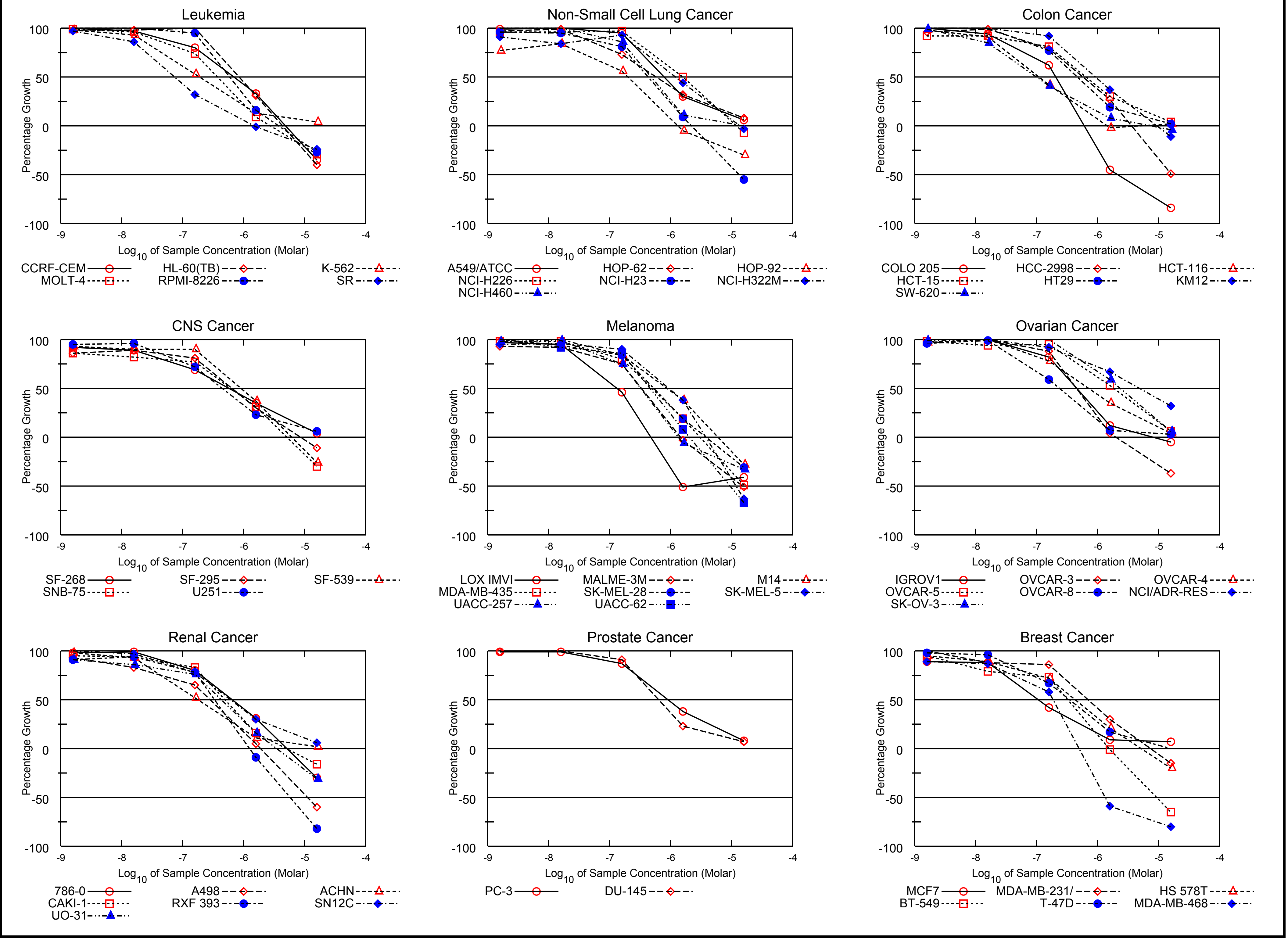

National Cancer Institute Developmental Therapeutics Program
In-Vitro Testing Results

NSC : D - 763852 / 1	Experiment ID : 1203NS37	Test Type : 08	Units : Molar
Report Date : July 29, 2012	Test Date : March 26, 2012	QNS :	MC :
COMI : CNX026_1062_VN (115656)	Stain Reagent : SRB Dual-Pass Related	SSPL : 075T	

			Log10 Concentration												
	Time		Mean Optical Densities					Percent Growth							
Panel/Cell Line	Zero	Ctrl	-8.8	-7.8	-6.8	-5.8	-4.8	-8.8	-7.8	-6.8	-5.8	-4.8	GI50	TGI	LC50
**Leukemia**															
CCRF-CEM	0.709	2.530	2.521	2.472	2.159	1.308	0.462	100	97	80	33	-35	6.46E-7	4.58E-6	> 1.50E-5
HL-60(TB)	0.902	2.465	2.437	2.437	2.558	1.388	0.544	98	98	106	31	-40	8.38E-7	4.12E-6	> 1.50E-5
K-562	0.273	1.679	1.657	1.583	1.023	0.452	0.326	98	93	53	13	4	1.81E-7	> 1.50E-5	> 1.50E-5
MOLT-4	0.728	2.293	2.274	2.237	1.892	0.872	0.520	99	96	74	9	-29	3.54E-7	2.62E-6	> 1.50E-5
RPMI-8226	0.911	2.378	2.437	2.418	2.302	1.139	0.663	104	103	95	16	-27	5.51E-7	3.46E-6	> 1.50E-5
SR	0.453	1.398	1.366	1.268	0.756	0.447	0.345	97	86	32	-1	-24	7.00E-8	1.36E-6	> 1.50E-5
**Non-Small Cell Lung Cancer**															
A549/ATCC	0.256	1.105	1.095	1.153	1.062	0.514	0.309	99	106	95	30	6	7.45E-7	> 1.50E-5	> 1.50E-5
HOP-62	0.332	0.979	0.962	0.972	0.806	0.540	0.382	97	99	73	32	8	5.51E-7	> 1.50E-5	> 1.50E-5
HOP-92	1.339	1.653	1.580	1.603	1.514	1.278	0.932	77	84	56	-5	-30	1.86E-7	1.26E-6	> 1.50E-5
NCI-H226	0.563	1.104	1.085	1.081	1.091	0.833	0.526	96	96	97	50	-7	1.49E-6	1.15E-5	> 1.50E-5
NCI-H23	0.598	1.723	1.677	1.671	1.512	0.695	0.271	96	95	81	9	-55	4.04E-7	2.05E-6	1.26E-5
NCI-H322M	0.849	1.598	1.533	1.480	1.549	1.180	0.820	91	84	93	44	-3	1.14E-6	1.27E-5	> 1.50E-5
NCI-H460	0.255	2.101	2.208	2.226	1.843	0.456	0.261	106	107	86	11	.	4.52E-7	> 1.50E-5	> 1.50E-5
**Colon Cancer**															
COLO 205	0.321	1.090	1.124	1.045	0.797	0.176	0.051	104	94	62	-45	-84	1.94E-7	5.67E-7	1.98E-6
HCC-2998	0.364	1.064	1.036	1.061	0.917	0.552	0.184	96	99	79	27	-49	5.39E-7	3.37E-6	> 1.50E-5
HCT-116	0.227	1.600	1.570	1.473	0.805	0.223	0.261	98	91	42	-2	2	1.03E-7	.	> 1.50E-5
HCT-15	0.311	1.743	1.625	1.634	1.475	0.746	0.362	92	92	81	30	4	6.17E-7	> 1.50E-5	> 1.50E-5
HT29	0.223	1.193	1.201	1.194	0.969	0.412	0.247	101	100	77	19	2	4.41E-7	> 1.50E-5	> 1.50E-5
KM12	0.443	1.964	1.986	2.012	1.837	1.007	0.396	101	103	92	37	-11	8.69E-7	8.99E-6	> 1.50E-5
SW-620	0.288	1.762	1.740	1.542	0.899	0.402	0.277	99	85	41	8	-4	9.55E-8	6.99E-6	> 1.50E-5
**CNS Cancer**															
SF-268	0.605	1.778	1.680	1.644	1.415	1.012	0.652	92	89	69	35	4	5.37E-7	> 1.50E-5	> 1.50E-5
SF-295	0.963	2.371	2.180	2.212	2.097	1.385	0.858	86	89	81	30	-11	6.02E-7	8.12E-6	> 1.50E-5
SF-539	0.954	2.321	2.230	2.178	2.182	1.464	0.703	93	90	90	37	-26	8.61E-7	5.78E-6	> 1.50E-5
SNB-75	0.687	1.304	1.220	1.195	1.163	0.878	0.484	86	82	77	31	-30	5.81E-7	4.87E-6	> 1.50E-5
U251	0.326	1.370	1.317	1.326	1.079	0.562	0.385	95	96	72	23	6	4.19E-7	> 1.50E-5	> 1.50E-5
**Melanoma**															
LOX IMVI	0.300	1.790	1.740	1.723	0.988	0.149	0.178	97	95	46	-51	-41	1.25E-7	4.50E-7	.
MALME-3M	0.686	1.140	1.109	1.104	1.028	0.663	0.334	93	92	75	-3	-51	3.15E-7	1.36E-6	1.41E-5
M14	0.454	1.407	1.433	1.345	1.256	0.817	0.326	103	94	84	38	-28	8.28E-7	5.62E-6	> 1.50E-5
MDA-MB-435	0.461	1.719	1.693	1.696	1.462	0.697	0.234	98	98	80	19	-49	4.60E-7	2.83E-6	> 1.50E-5
SK-MEL-28	0.371	0.956	0.986	0.972	0.862	0.480	0.256	105	103	84	19	-31	4.96E-7	3.55E-6	> 1.50E-5
SK-MEL-5	0.681	2.302	2.222	2.238	2.144	1.297	0.255	95	96	90	38	-63	8.84E-7	3.58E-6	1.12E-5
UACC-257	0.558	1.135	1.124	1.128	0.990	0.524	0.375	98	99	75	-6	-33	3.04E-7	1.26E-6	> 1.50E-5
UACC-62	0.917	2.077	2.120	1.982	1.914	1.012	0.302	104	92	86	8	-67	4.34E-7	1.93E-6	8.90E-6
**Ovarian Cancer**															
IGROV1	0.652	1.657	1.675	1.662	1.472	0.775	0.619	102	100	82	12	-5	4.28E-7	7.58E-6	> 1.50E-5
OVCAR-3	0.521	1.514	1.536	1.525	1.393	0.562	0.328	102	101	88	4	-37	4.25E-7	1.89E-6	> 1.50E-5
OVCAR-4	0.418	0.816	0.804	0.821	0.729	0.559	0.435	97	101	78	35	4	6.80E-7	> 1.50E-5	> 1.50E-5
OVCAR-5	0.453	1.198	1.187	1.154	1.164	0.846	0.501	98	94	95	53	6	1.72E-6	> 1.50E-5	> 1.50E-5
OVCAR-8	0.282	1.076	1.042	1.067	0.751	0.337	0.307	96	99	59	7	3	2.24E-7	> 1.50E-5	> 1.50E-5
NCI/ADR-RES	0.393	1.381	1.382	1.379	1.305	1.058	0.706	100	100	92	67	32	4.59E-6	> 1.50E-5	> 1.50E-5
SK-OV-3	0.531	1.100	1.095	1.124	1.135	0.867	0.568	99	104	106	59	6	2.22E-6	> 1.50E-5	> 1.50E-5
**Renal Cancer**															
786-0	0.774	2.242	2.211	2.226	1.945	1.230	0.543	98	99	80	31	-30	6.12E-7	4.85E-6	> 1.50E-5
A498	1.111	1.640	1.602	1.552	1.454	1.139	0.444	93	83	65	5	-60	2.65E-7	1.80E-6	1.05E-5
ACHN	0.344	1.369	1.344	1.298	0.881	0.458	0.361	98	93	52	11	2	1.71E-7	> 1.50E-5	> 1.50E-5
CAKI-1	0.809	2.096	2.032	2.019	1.875	1.015	0.680	95	94	83	16	-16	4.65E-7	4.74E-6	> 1.50E-5
RXF 393	0.682	1.062	1.028	1.039	0.979	0.620	0.122	91	94	78	-9	-82	3.15E-7	1.18E-6	5.44E-6
SN12C	0.545	1.736	1.741	1.697	1.475	0.908	0.618	100	97	78	30	6	5.83E-7	> 1.50E-5	> 1.50E-5
UO-31	0.897	1.964	1.868	1.817	1.712	1.071	0.621	91	86	76	16	-31	4.12E-7	3.33E-6	> 1.50E-5
**Prostate Cancer**															
PC-3	0.466	1.609	1.603	1.597	1.460	0.904	0.561	99	99	87	38	8	8.63E-7	> 1.50E-5	> 1.50E-5
DU-145	0.365	1.332	1.321	1.360	1.242	0.586	0.431	99	103	91	23	7	5.97E-7	> 1.50E-5	> 1.50E-5
**Breast Cancer**															
MCF7	0.330	1.691	1.538	1.527	0.902	0.451	0.419	89	88	42	9	7	1.00E-7	> 1.50E-5	> 1.50E-5
MDA-MB-231/ATCC	0.599	1.134	1.143	1.069	1.058	0.762	0.508	102	88	86	30	-15	6.65E-7	6.97E-6	> 1.50E-5
HS 578T	0.853	1.488	1.454	1.418	1.313	0.989	0.679	95	89	72	21	-20	4.13E-7	4.87E-6	> 1.50E-5
BT-549	0.908	1.788	1.744	1.601	1.554	0.899	0.314	95	79	73	-1	-65	3.10E-7	1.45E-6	8.64E-6
T-47D	0.558	1.244	1.231	1.216	1.017	0.673	0.561	98	96	67	17	.	3.26E-7	> 1.50E-5	> 1.50E-5
MDA-MB-468	0.551	1.246	1.171	1.155	0.957	0.226	0.111	89	87	58	-59	-80	1.77E-7	4.71E-7	1.26E-6

National Cancer Institute Developmental Therapeutics Program
Mean Graphs

NSC : D - 763852/1 | Units :Molar | SSPL :075T | EXP. ID :1203NS37

Report Date :July 29, 2012 | Test Date :March 26, 2012

Panel/Cell Line	Log_{10}GI50	Log_{10}TGI	Log_{10}LC50
**Leukemia**			
CCRF-CEM	-6.19	-5.34	> -4.82
HL-60(TB)	-6.08	-5.38	> -4.82
K-562	-6.74	> -4.82	> -4.82
MOLT-4	-6.45	-5.58	> -4.82
RPMI-8226	-6.26	-5.46	> -4.82
SR	-7.16	-5.87	> -4.82
**Non-Small Cell Lung Cancer**			
A549/ATCC	-6.13	> -4.82	> -4.82
HOP-62	-6.26	> -4.82	> -4.82
HOP-92	-6.73	-5.90	> -4.82
NCI-H226	-5.83	-4.94	> -4.82
NCI-H23	-6.39	-5.69	-4.90
NCI-H322M	-5.94	-4.90	> -4.82
NCI-H460	-6.34	> -4.82	> -4.82
**Colon Cancer**			
COLO 205	-6.71	-6.25	-5.70
HCC-2998	-6.27	-5.47	> -4.82
HCT-116	-6.99		> -4.82
HCT-15	-6.21	> -4.82	> -4.82
HT29	-6.36	> -4.82	> -4.82
KM12	-6.06	-5.05	> -4.82
SW-620	-7.02	-5.16	> -4.82
**CNS Cancer**			
SF-268	-6.27	> -4.82	> -4.82
SF-295	-6.22	-5.09	> -4.82
SF-539	-6.07	-5.24	> -4.82
SNB-75	-6.24	-5.31	> -4.82
U251	-6.38	> -4.82	> -4.82
**Melanoma**			
LOX IMVI	-6.90	-6.35	
MALME-3M	-6.50	-5.87	-4.85
M14	-6.08	-5.25	> -4.82
MDA-MB-435	-6.34	-5.55	> -4.82
SK-MEL-28	-6.30	-5.45	> -4.82
SK-MEL-5	-6.05	-5.45	-4.95
UACC-257	-6.52	-5.90	> -4.82
UACC-62	-6.36	-5.72	-5.05
**Ovarian Cancer**			
IGROV1	-6.37	-5.12	> -4.82
OVCAR-3	-6.37	-5.72	> -4.82
OVCAR-4	-6.17	> -4.82	> -4.82
OVCAR-5	-5.76	> -4.82	> -4.82
OVCAR-8	-6.65	> -4.82	> -4.82
NCI/ADR-RES	-5.34	> -4.82	> -4.82
SK-OV-3	-5.65	> -4.82	> -4.82
**Renal Cancer**			
786-0	-6.21	-5.31	> -4.82
A498	-6.58	-5.74	-4.98
ACHN	-6.77	> -4.82	> -4.82
CAKI-1	-6.33	-5.32	> -4.82
RXF 393	-6.50	-5.93	-5.26
SN12C	-6.23	> -4.82	> -4.82
UO-31	-6.38	-5.48	> -4.82
**Prostate Cancer**			
PC-3	-6.06	> -4.82	> -4.82
DU-145	-6.22	> -4.82	> -4.82
**Breast Cancer**			
MCF7	-7.00	> -4.82	> -4.82
MDA-MB-231/ATCC	-6.18	-5.16	> -4.82
HS 578T	-6.38	-5.31	> -4.82
BT-549	-6.51	-5.84	-5.06
T-47D	-6.49	> -4.82	> -4.82
MDA-MB-468	-6.75	-6.33	-5.90
_MID	-6.35	-5.28	-4.88
Delta	0.81	1.07	1.02
Range	1.82	1.53	1.08

Scale: +3 +2 +1 0 -1 -2 -3
